# Reduction in pericyte coverage leads to blood–brain barrier dysfunction via endothelial transcytosis following chronic cerebral hypoperfusion

**DOI:** 10.1186/s12987-021-00255-2

**Published:** 2021-05-05

**Authors:** Zhengyu Sun, Chenhao Gao, Dandan Gao, Ruihua Sun, Wei Li, Fengyu Wang, Yanliang Wang, Huixia Cao, Guoyu Zhou, Jiewen Zhang, Junkui Shang

**Affiliations:** 1 Department of Neurology, Henan Provincial People’s Hospital, Zhengzhou University People’s Hospital, Henan University People’s Hospital, Zhengzhou, 450003 Henan China; 2 Department of Nephrology, Henan Provincial Key Laboratory of Kidney Disease and Immunology, Henan Provincial People’s Hospital, Zhengzhou University People’s Hospital, Henan University People’s Hospital, Zhengzhou, 450003 Henan China; 3 School of Public Health, Zhengzhou University, Zhengzhou, 450001 Henan China

**Keywords:** Cerebral small vessel disease, Chronic cerebral hypoperfusion, BBB permeability, BBB protection, Pericyte, Endothelial transcytosis, White matter lesions, TGF-β signaling

## Abstract

**Background:**

Chronic cerebral hypoperfusion (CCH) is the leading cause of cerebral small vessel disease (CSVD). CCH is strongly associated with blood–brain barrier (BBB) dysfunction and white matter lesions (WMLs) in CSVD. However, the effects of CCH on BBB integrity and components and the cellular and molecular mechanisms underlying the effects of BBB dysfunction remain elusive. Whether maintaining BBB integrity can reverse CCH-induced brain damage has also not been explored.

**Methods:**

In this study, we established a rat model of CSVD via permanent bilateral common carotid artery occlusion (2VO) to mimic the chronic hypoperfusive state of CSVD. The progression of BBB dysfunction and components of the BBB were assessed using immunostaining, Western blotting, transmission electron microscopy (TEM) and RNA sequencing. We also observed the protective role of imatinib, a tyrosine kinase inhibitor, on BBB integrity and neuroprotective function following CCH. The data were analyzed using one-way or two-way ANOVA.

**Results:**

We noted transient yet severe breakdown of the BBB in the corpus callosum (CC) following CCH. The BBB was severely impaired as early as 1 day postoperation and most severely impaired 3 days postoperation. BBB breakdown preceded neuroinflammatory responses and the formation of WMLs. Moreover, pericyte loss was associated with BBB impairment, and the accumulation of serum protein was mediated by increased endothelial transcytosis in the CC. RNA sequencing also revealed increased transcytosis genes expression. BBB dysfunction led to brain damage through regulation of TGF-β/Smad2 signaling. Furthermore, imatinib treatment ameliorated serum protein leakage, oligodendrocyte progenitor cell (OPC) activation, endothelial transcytosis, microglial activation, and aberrant TGF-β/Smad2 signaling activation.

**Conclusions:**

Our results indicate that reduced pericyte coverage leads to increased BBB permeability via endothelial transcytosis. Imatinib executes a protective role on the BBB integrity via inhibition of endothelial transcytosis. Maintenance of BBB integrity ameliorates brain damage through regulation of TGF-β/Smad2 signaling following CCH; therefore, reversal of BBB dysfunction may be a promising strategy for CSVD treatment.

**Supplementary Information:**

The online version contains supplementary material available at 10.1186/s12987-021-00255-2.

## Introduction

Chronic cerebral hypoperfusion (CCH) and blood–brain barrier (BBB) dysfunction are two significant pathological features of the aging brain [1–3]. Older age is the single most important risk factor for cerebral small vessel disease (CSVD) [4]. CSVD is one of the most common causes of vascular dementia (VD) [5]. VD is a neurodegenerative disease that is second only to Alzheimer’s disease (AD) in prevalence [6]. CSVD imposes a serious burden on the development of society. The pathogenesis of CSVD has not been clearly established. Although several pathological changes, including CCH, BBB impairment, oxidative stress, inflammation and white matter hyperintensities (WMHs), have been shown to be related to CSVD [7], the cascade of pathological changes that occur in CSVD is still not fully understood. Therefore, we approached this topic by exploring the cellular and molecular mechanisms that regulate the relationship between CCH and BBB function.

The adult brain relies mostly on the continuous influx of glucose from the blood for energy. The rates of glucose and oxygen metabolic decrease in normal aging and are further exacerbated in AD. CCH alters cerebral blood flow (CBF) and brain energy metabolism. Metabolic alterations strongly influence the progression of AD and VD [8, 9]. CCH has also been suggested to cause of BBB dysfunction and WMHs [10]. By restricting the free diffusion of circulating toxins or pathogens, the BBB provides a homeostatic brain microenvironment for healthy neural function [11, 12]. Cross-sectional studies have revealed that CCH is correlated with BBB impairment. CCH is also related to the severity of WMHs [10]. BBB impairment is more severe in areas near WMHs than in areas of apparently normal white matter (WM) in CSVD [10, 13]. This indicates that BBB impairment is a key factor linking CCH and WMHs in CSVD.

BBB integrity is maintained by endothelial cell (EC), pericyte, astrocyte, microglia, tight junction (TJ) and extracellular basement membrane (BM) [14]. BBB components form a complex, dynamic structure, and BBB impairment therefore involves these various components [15, 16]. The precise responses of all BBB components to CCH have not been thoroughly characterized. Meanwhile, it is unclear whether BBB breakdown is the primary cause of brain parenchyma damage following CCH or secondary to this damage. Furthermore, BBB breakdown leads to inflammation, oxidative stress, neural injury, loss of neuronal connectivity and neurodegeneration [17]. However, whether ameliorating BBB disruption executes a protective role on neural function following CCH is still poorly understood.

Bilateral common carotid artery occlusion (2VO) is used in rats to mimic the chronic hypoperfusive state of CSVD, and rat subjected to 2VO are used as animal model to assess the mechanisms of CSVD [18]. Based on the evidence presented above, we first used the 2VO rat model in this study to determine the effects of CCH on changes in BBB permeability and BBB components and brain parenchyma damage. It has been demonstrated that imatinib, a tyrosine kinase inhibitor, counteracts brain oedema, stroke lesion volume and haemorrhagic transformation in rodent stroke model [19]. Secondly, we observed the BBB protective role of imatinib following CCH. Lastly, we explored the molecular mechanisms that regulate neural injury after BBB breakdown and determined whether BBB impairment is the key pathophysiological mechanism following CCH.

Our results indicate that BBB impairment occurs early in the disease process, preceding neuroinflammatory responses and white matter lesions (WMLs). The mechanism of BBB disruption appears to be pericyte loss. Toxins are able to enter the brain parenchyma due to the increased endothelial transcytosis after BBB impairment. Imatinib can ameliorate BBB disruption via suppression of endothelial transcytosis. Maintenance of BBB integrity via imatinib alleviates oligodendrocyte progenitor cell (OPC) activation, microglial activation, and aberrant TGF-β/Smad2 signaling activation. Imatinib shows neuroprotective function. This study helps explain the effects of BBB injury following CCH and identifies a new potential therapeutic target for BBB integrity maintenance, providing a theoretical basis for the development of targeted treatment strategies.

## Material and methods

### Animals

Adult male Sprague Dawley rats (weighing 280–300 g, aged 8–12 weeks) selected for this study were housed at a temperature of 24–26 °C on a 12-h light/dark cycle with free access to food and water. Forty-eight rats were used for immunohistochemistry, which was performed to assess serum protein leakage and changes in BBB components in the corpus callosum (CC) at various time intervals following CCH. Thirty rats were injected with Evans blue (EB) dye via the tail vein and used for the assessment of BBB permeability, protein extraction and transmission electron microscopy(TEM). Twenty rats were used to measure CBF. Eighty rats were used to assess BBB protection role of imatinib. Twenty rats were used for TEM after imatinib treatment. All experimental procedures were approved by and performed in accordance with the standards of the Experimental Animal Committee of Henan University and Henan Provincial People’s Hospital.

### Establishment of the CCH model

As previously described [20], CCH was induced in the rats by 2VO. In brief, the surgical procedure was performed under sterile conditions. The rats were anesthetized via intraperitoneal (i.p.) injection of a combination of ketamine (50 mg/kg) and xylazine (10 mg/kg) and placed ventral side up. A 2-cm-long midline incision was made on the ventral side of the cervical neck of each rat. Following careful separation of muscle tissue, nerves and other adjacent tissue, the bilateral common carotid arteries were identified and permanently ligated using a silk ligature. For the sham operation, the same procedure was performed to expose the common carotid arteries, but no ligation was performed. Afterward, the muscle tissue and skin were sutured in layers. Finally, postoperative rats were placed on a warm blanket for recovery.

### Measurement of regional CBF

Rats were anesthetized and fixed on a stereotaxic frame (RWD Lifescience, China). Under sterile conditions, a bone window was made at 1-1.5 mm anterior to the bregma and 0-3 mm left to the midline. A laser Doppler flowmetry (Moor Instruments, UK) probe was fixed to the center of the bone window and CBF was recorded 1, 3, 7, and 28 days postoperation.

### Measurement of brain water content and BBB permeability

Brain water content and BBB permeability were examined 1, 3, 7, and 28 days postoperation. EB extravasation was used to assess BBB permeability [21]. In brief, 2% EB (3 mL/kg, Sigma) was injected via the tail vein at various timepoints as indicated. After the EB was allowed to circulate for 2 h, the rats were anesthetized and then perfused transcardially with normal saline solution. The whole brains were collected and divided into the left and right hemispheres. The left hemisphere was used for the measurement of brain water content. The right hemisphere was further cut into 1-mm-thick sections using a stainless steel rat brain matrices (RWD Life Science, China). One section was used for Western blotting, and one section was used for TEM. The other sections were used to assess EB extravasation. To measure brain water content, the left hemisphere was weighed before and after being dehydrated in an oven for 24 h at 100 ℃. The wet/dry brain weight ratio was used to quantify brain water content for statistical analysis. For EB extravasation assessment, sections of the right hemisphere were weighed, homogenized in 1 ml of 50% trichloroacetic acid, and then centrifuged at 10,000 rpm for 30 min. The supernatant was collected and mixed with an equal volume of ethanol. The concentration of EB was determined by spectrophotometry at an absorbance of 620 nm. EB content (μg/g) was calculated according to the standard curve to evaluate BBB permeability.

### Histology and immunohistochemistry

At different timepoints after operation, rats were anesthetized and perfused transcardially with 100 ml normal saline solution, followed by 500 ml phosphate-buffered fixative solution composed of 4% paraformaldehyde (PFA, pH 7.4). Next, the brains were removed, postfixed overnight, and finally cryoprotected in phosphate-buffered sucrose (30%) for 3–5 days. Frozen sections (20 µm) were prepared using a cryostat (Leica) and processed for histological examination. Immunohistochemistry staining was performed as previously described [22, 23]. The following primary antibodies were used: rabbit anti-Olig2 (1:200, Millipore); mouse anti-PCNA (1:200, Invitrogen); rabbit anti-collagen IV (COIV, 1:200, Abcam); chicken anti-albumin (ALB, 1:500, Abcam); mouse anti-immunoglobulin G (IgG, 1:200, Jackson ImmunoResearch); mouse anti-platelet-derived growth factor receptor beta (PDGFR-β, 1:200, Abcam); rabbit anti-desmin (1:100, Cell Signaling Technology); mouse anti-Glut1 (1:200, Abcam); mouse anti-GFAP (1:200, Sigma); rabbit anti-Iba1 (1:300, Wako); rabbit anti-phosphorylated Smad2 (pSmad2, 1:500, Millipore); and mouse anti-myelin basic protein (MBP, 1:200, Biolegend). Alexa Fluor 488- and 594- conjugated goat secondary antibodies (1:500, Thermo Fisher) were also used. Nuclear staining was performed using 4’,6’-diamidino-2-phenylindole dihydrochloride (DAPI, 1:2000, Thermo Fisher). The sections were examined using a confocal laser scanning microscope (TCS SP8, Leica, German).

### Western blotting

To determine the change in protein levels in the CC, CC tissue was precisely isolated from the right hemisphere closed to 1.0 mm anterior to the bregma on ice. Once weighed, the tissue was digested in RIPA lysis buffer and homogenized. The protein concentration was quantified, and then the protein were separated on 10% SDS–PAGE gels and transferred to nitrocellulose membranes (Invitrogen, USA). After three times washes in TBS with 0.05% Tween-20 (TBST), the membranes were blocked in TBST with 5% skim milk for 2 h at room temperature. The membranes were incubated with primary antibodies at 4 ℃ overnight and then further incubated with HRP-conjugated secondary antibodies (1:2000) for 1 h at room temperature. The following primary antibodies were used: mouse anti-PDGFR-β (1:1000, Abcam); rabbit anti-TGF-β1 (1:2000, Abcam); rabbit anti-pSmad2 (1:1000, Millipore); rabbit anti-occludin (1:2000, Thermo Fisher); rabbit anti-claudin 5 (1:2000, Thermo Fisher); rabbit anti-ZO-1 (1:1000, Thermo Fisher) and rabbit anti-MBP (1:2000, Abcam). Western Bright ECL solution was used to develop the blots, which were analyzed using GelPro Analyzer 6.0 software (Media Cybernetics, Rockville, MD, USA).

### TEM

After the right hemisphere was divided into 1-mm sections, tissue closed to the bregma was selected and further cut into 1 × 1 × 1-mm tissue blocks. The tissue blocks were incubated with 2.5% glutaraldehyde for 6 h, dehydrated and embedded in epoxy resin. Ultrathin sections were cut at 60 nm thickness and observed under an electron microscope (Hitachi TEM system, Japan).

### RNA extraction

Using stainless steel rat brain matrices (RWD Life Science, China), the whole brains were cut into 1-mm-thick section. The sections closed to 1-1.5 mm anterior to the bregma was selected. The CC was precisely dissected and collected in TRIzol for RNA sequencing. The CC of three rats was collected together as one biological replicates. Three biological replicates were used in the Sham, 1 day, 3 day and 7 day groups respectively.

### RNA sequencing and data analysis

Total RNA was extracted in the CC to perform RNA sequencing analysis. The products were sequenced using Illumina HiSeq™ 4000 by Gene Denovo Biotechnology Co. (Guangzhou, China). RNA sequencing raw reads were mapped to the reference genome using HISAT2 [24] (version 2.1.0). Differentially expressed genes were identified using DESeq2 [25] based on the criteria that P value < 0.05 and |log_2_Fold Change|> 1.

### BBB protection

Imatinib inhibits signaling of platelet-derived growth factor receptor (PDGFR) by inducing receptor dimerization via binding to RTK phosphorylation sites [26]. Imatinib has been found to maintain BBB integrity [27]. After CCH, rats were administered imatinib (150 mg/kg) by i.p. injections every 12 h for 3 days. The lesion control rats were given normal saline after CCH. After the final injection, rats were euthanized and perfused.

### Cell counts

To quantify the number of various cell types in the CC, five fields were randomly chosen from each section and imaged under a confocal microscope (TCS SP8, Leica, German) with a 40 × or 63 × oil immersion objective in 10–12 µm thick z-stacks. A total of five sections were analyzed. Every cell expressing the selected marker was manually counted using Image-Pro Plus 7 (Media Cybernetics, USA). The data are presented as the average cell number in a single field per section.

### Quantification of vessel diameter and pericyte coverage

Confocal images were acquired under a 40 × objective. Using Image-Pro Plus 7, the Glut1-positive vessel diameter in each image was measured manually. The COIV-positive brain capillary length and PDGFR-β-positive pericyte length were measured manually using Image-Pro Plus 7. The ratio of PDGFR-β-positive pericyte length to COIV-positive brain capillary length was calculated and coverted to percentage as an indicator of pericyte coverage.

### Statistical analysis

Comparisons between multiple group comparisons were made by one-way ANOVA followed by Dunnett’s post hoc test or two-way ANOVA. Data normality was assessed using the Shapiro–Wilk test. The data are presented as the mean ± SD, and the boxplots show the maximum and minimum values. Statistical analysis was performed and graphs were made using GraphPad Prism 8.0 software. Values were considered significant at p < 0.05.

## Results

### CBF changes following CCH

**Fig. 1:**
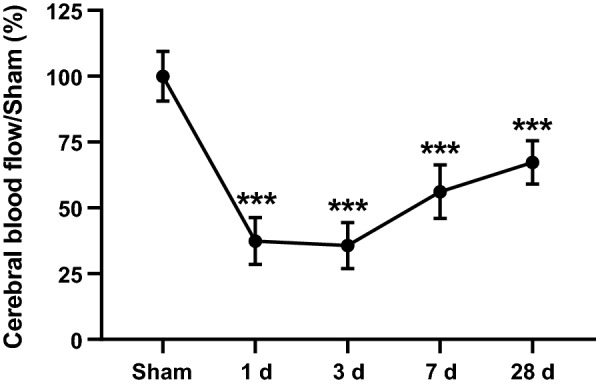
The temporal profile of CBF following CCH. ***p < 0.001 compared to the Sham group; one-way ANOVA followed by Dunnett’s post hoc test

Quantification of CBF revealed progressive CBF reduction from 1 to 3 days postoperation (percentage of Sham: 100 ± 9.4, 37 ± 8.9 and 36 ± 8.7 in the Sham, 1 day and 3 day groups, respectively). The greatest reduction of CBF was 3 days postoperation. The CBF started to gradually recover 7 days postoperation (percentage of Sham: 56 ± 10.1 in the 7 day group). However, there was still significantly lower the 28 day group (percentage of Sham: 67 ± 8.2 in the 28 day group) compared to the Sham group (Fig. 1).

### Transient and severe BBB breakdown following CCH

**Fig. 2:**
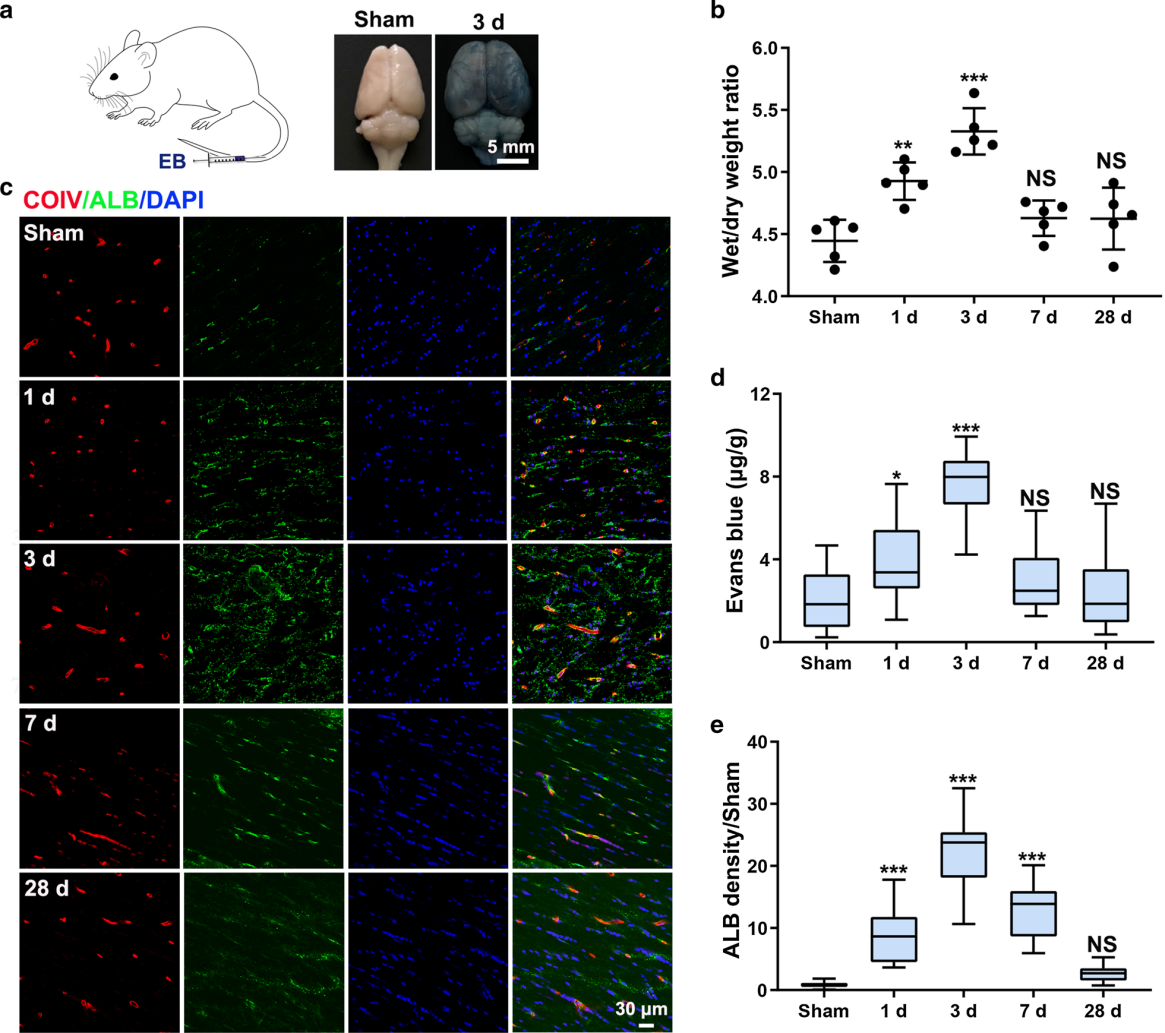
Increased BBB permeability following CCH. **a** Left: schematic illustrating the injection of EB into the tail vein; right: gross anatomic changes in the Sham group and the 3 day group after EB injection. **b** Time-dependent change in the brain wet/dry weight ratio after operation. **c** Triple staining for COIV (red), ALB (green) and DAPI (blue) for assessment of ALB leakage in the CC. **d, e** Quantification of EB and ALB staining at different timepoints. n = 5 per group; NS, not significant; *p < 0.05, **p < 0.01 and ***p < 0.001 compared to the Sham group; one-way ANOVA followed by Dunnett’s post hoc test

Central nervous system (CNS) homeostasis is dependent on the integrity of the BBB. The BBB prevents dysregulated transit of molecules into the brain and very effectively blocks toxins and pathogens to preserve delicate neural functioning [28]. The dynamic changes in BBB integrity and the cascade reaction that occur following CCH are still largely unclear. We began by examining the change in BBB permeability following CCH. Increased brain water content indicates increased BBB permeability. We found that brain water content was increased 1 day postoperation and the brain edema was most severe 3 days postoperation (wet/dry weight ratio: 4.45 ± 0.17, 4.93 ± 0.15 and 5.33 ± 0.19 in the Sham, 1 day and 3 day groups, respectively) (Fig. 2a, b). Recovery began 7 days postoperation (wet/dry weight ratio: 4.63 ± 0.14 in the 7 day group), and brain water content had nearly returned to the normal level by 28 days postoperation (wet/dry weight ratio: 4.63 ± 0.25 in the 28 day group) (Fig. 2b). Measurement of dye leakage following injection of EB into the tail vein was also used to assess BBB integrity. As expected, EB accumulated in the CC following CCH. EB extravasation was apparent on gross examination of the brain 3 days postoperation (Fig. 2a). The change in EB accumulation mirrored the change in brain water content (EB concentration: 2.08 ± 1.38, 4.09 ± 1.79, 7.68 ± 1.45, 3.02 ± 1.54 and 2.42 ± 1.89 μg/g in the Sham, 1 day, 3 day, 7 day and 28 day groups, respectively) (Fig. 2d). Quantification of EB accumulation also revealed BBB impairment in the cortex and striatum (cortex EB concentration: 1.92 ± 1.07, 7.34 ± 2.26, and striatum EB concentration: 1.62 ± 0.90, 6.02 ± 1.95 μg/g in the Sham and 3 day groups, respectively) (Additional file 1: Figure S1). These results suggest that severe global barrier leakage occurs as early as 1 day following CCH and that spontaneous recovery occurs 7 days postoperation thereafter.

After BBB breakdown, endogenous circulating macromolecules, which may be toxic to neurons, leak into the brain. Immunostaining revealed significant leakage of the serum protein ALB outside vessels in the CC following CCH. ALB leakage was most severe 3 days postoperation (ALB density/Sham: 0.89 ± 0.45, 8.74 ± 4.34, 22.70 ± 5.66, 12.75 ± 4.41 and 2.64 ± 1.16 in the Sham, 1 day, 3 day, 7 day and 28 day groups, respectively) (Fig. 2c, e). We then examined plasma-derived IgG deposition in the CC. Immunostaining revealed some IgG within vessels and some IgG leakage outside vessels (Additional file 1: Figure S2A). Total IgG levels were increased 3 days postoperation (total IgG density/Sham: 0.92 ± 0.41, 1.46 ± 0.63, 1.75 ± 0.53, 1.00 ± 0.34 and 1.04 ± 0.38 in the Sham, 1 day, 3 day, 7 day and 28 day groups, respectively) (Additional file 1: Figure S2B). IgG leakage was significantly increased 1 day postoperation, reaching the most severe level 3 days postoperation (outside IgG density/Sham: 2.16 ± 1.16, 7.56 ± 2.21, 29.17 ± 7.60, 5.89 ± 2.26 and 5.19 ± 2.17 in the Sham, 1 day, 3 day, 7 day and 28 day groups, respectively) (Additional file 1: Figure S2C).

### Reduction in pericyte coverage leads to BBB dysfunction following CCH

**Fig. 3:**
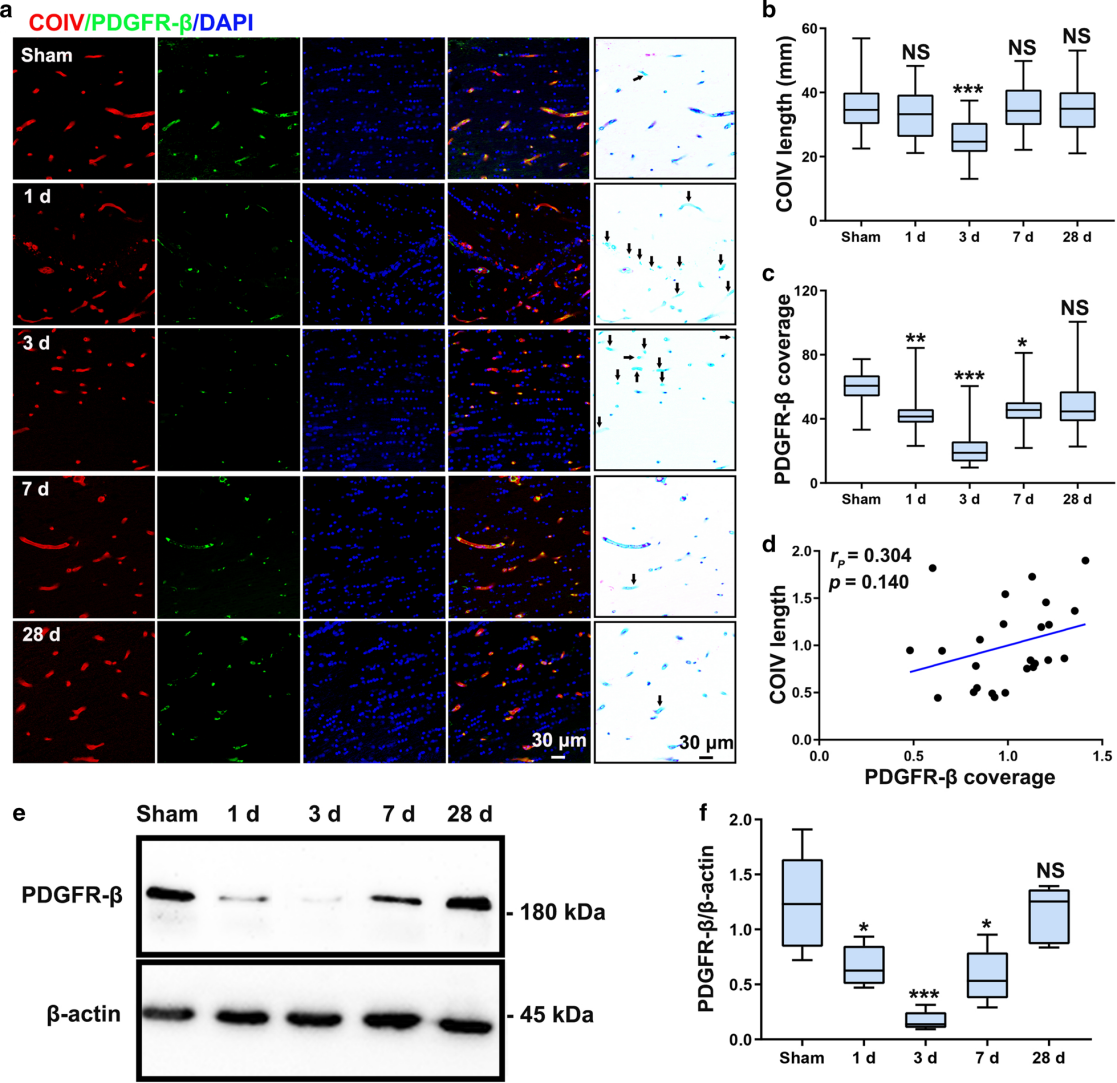
Pericyte loss in the CC following CCH. **a** Triple staining for COIV (red), PDGFR-β (green) and DAPI (blue) for assessment of pericyte coverage of brain capillaries in the CC. A schematic of pericyte coverage loss in capillaries, as seen in a representative confocal microscopy image, is shown in the right column. The black arrows indicate pericyte coverage loss. **b, c** Quantification of COIV-positive capillary length and pericyte coverage in the CC at different timepoints. **d** Correlation between capillary length and loss of pericyte coverage in the CC at 3 days postoperation. **e, f** Western blotting analysis and quantification of PDGFR-β expression in the CC at different timepoints. n = 8 per group; NS, not significant; *p < 0.05, **p < 0.01 and ***p < 0.001 compared to the Sham group; one-way ANOVA followed by Dunnett’s post hoc test

**Fig. 4:**
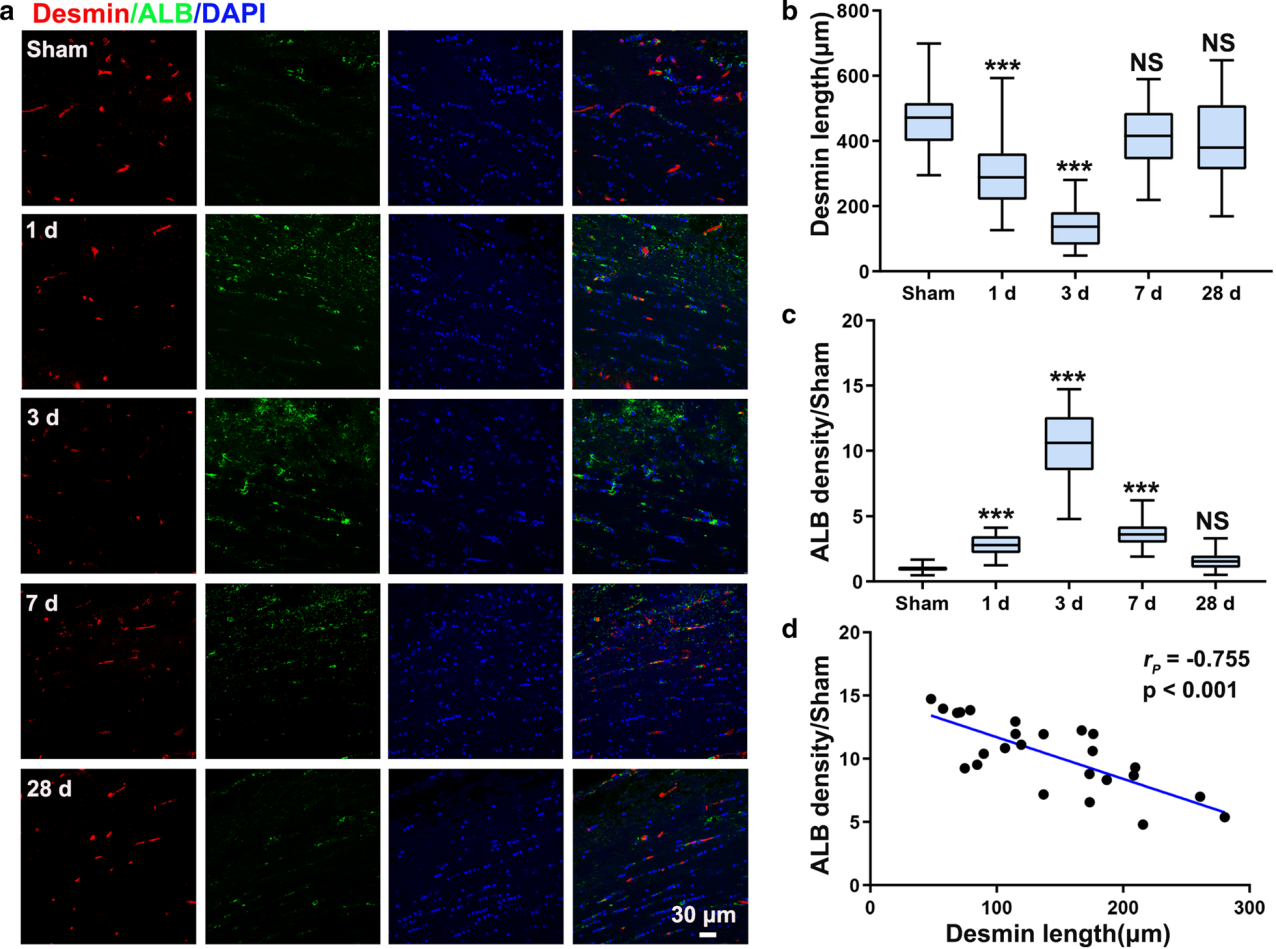
Correlation between pericyte loss and BBB leakage in the CC following CCH. **a** Triple staining for desmin (red), ALB (green) and DAPI (blue) for evaluation of the relationship between pericyte loss and BBB impairment in the CC following CCH. **b, c** Quantification of desmin-positive pericyte length and ALB density in the CC at different timepoints. **d** Correlation analysis of pericyte length and ALB density in the CC at 3 days postoperation. n = 8 per group; NS, not significant; ***p < 0.001 compared to the Sham group; one-way ANOVA followed by Dunnett’s post hoc test

BBB integrity depends on the completeness of BBB structure. We therefore studied the effects of CCH on BBB components. Pericytes play an important role in BBB function [27, 29]. Using dual immunostaining for PDGFR-β- and COIV-positive brain capillaries, we observed the initial changes in pericytes following CCH (Fig. 3). Pericyte coverage was significantly decreased in the 1 day group compared to the Sham group (Fig. 3a). Pericyte coverage loss reached the most severe level at 3 days postoperation, with a decrease of approximately 65% compared to the Sham group (Fig. 3a, c). However, by 7 days postoperation, pericyte coverage showed slight recovery. Pericyte coverage was restored to approximately 84% of that in the Sham group at 28 days postoperation, when pericyte coverage was not significantly different between the two groups (pericyte coverage: 60.13 ± 10.68, 43.81 ± 13.50, 20.97 ± 11.32, 46.12 ± 12.70 and 50.47 ± 19.01 in the Sham, 1 day, 3 day, 7 day and 28 day groups, respectively) (Fig. 3a, c). Capillary length was significantly reduced in the 3 day group compared to the Sham group (capillary length: 35.41 ± 8.03, 33.09 ± 7.92, 25.93 ± 6.46, 34.70 ± 7.16 and 34.12 ± 8.13 mm in the Sham, 1 day, 3 day, 7 day and 28 day groups, respectively) (Fig. 3a, b). Correlation analysis revealed that pericyte coverage was not correlated with capillary length (Fig. 3d), indicating that pericyte coverage loss was not due to capillary length reduction. Western blotting analysis also showed that PDGFR-β protein levels decreased from day 1 to day 3 postoperation and then increased from days 7 to 28 (Fig. 3e). PDGFR-β protein reduction was most severe 3 days postoperation (PDGFR-β/β-actin: 1.24 ± 0.45, 0.67 ± 0.19, 0.17 ± 0.09, 0.57 ± 0.25 and 1.14 ± 0.26 in the Sham, 1 day, 3 day, 7 day and 28 day groups, respectively) (Fig. 3f). Desmin is another pericyte marker. We sought to further confirm pericyte loss using desmin immunostaining, and we found that the pattern of desmin loss was indeed similar to that of PDGFR-β immunostaining (desmin length: 469.7 ± 100.3, 306.0 ± 112.1, 141.3 ± 64.2, 409.9 ± 91.0 and 414.4 ± 124.4 μm in the Sham, 1 day, 3 day, 7 day and 28 day groups, respectively) (Fig. 4). Interestingly, we found a significant negative correlation between pericyte coverage and ALB accumulation (Fig. 4d), indicating that pericyte loss is associated with BBB impairment.

Pericyte is not the only cell that regulates the permeability of the BBB [30]. EC, astrocyte and continuous complexes of endothelial junction are also integral components of the BBB [31]. To further elucidate the effect of pericyte loss on BBB impairment, we examined other components of the BBB. Glut1 is a marker of EC. Using Glut1 immunostaining to observe microvascular changes in the CC, we found no significant reduction in microvascular density (number of capillary: 20.25 ± 4.37, 20.75 ± 3.77, 18.38 ± 4.81, 21.50 ± 5.76 and 22.00 ± 6.09/section in the Sham, 1 day, 3 day, 7 day and 28 day groups, respectively) and an increase in microvascular diameter (diameter of capillary: 5.58 ± 1.58, 7.19 ± 2.22, 10.44 ± 3.20, 7.55 ± 1.67 and 4.57 ± 1.21 μm in the Sham, 1 day, 3 day, 7 day and 28 day groups, respectively) (Additional file 1: Figure S3). We then assessed the expression of TJ (i.e. occludin, claudin 5 and ZO-1) between ECs. The expression of occludin was downregulated in the 3 day group compared to the Sham group (occludin/β-actin: 0.57 ± 0.15, 0.47 ± 0.08, 0.34 ± 0.14, 0.50 ± 0.07 and 0.52 ± 0.16 in the Sham, 1 day, 3 day, 7 day and 28 day groups, respectively). However, there was no significant downregulation of claudin 5 expression (claudin 5/β-actin: 1.27 ± 0.24, 1.26 ± 0.41, 1.05 ± 0.11, 1.17 ± 0.24 and 1.22 ± 0.22 in the Sham, 1 day, 3 day, 7 day and 28 day groups, respectively) and ZO-1 expression (ZO-1/β-actin: 17.57 ± 2.63, 19.44 ± 3.57, 18.60 ± 3.51, 19.34 ± 3.17 and 18.05 ± 2.54 in the Sham, 1 day, 3 day, 7 day and 28 day groups, respectively) following CCH (Additional file 1: Figure S4).

Astrocytic coverage of blood vessels is also vital for BBB integrity [32]. Although the number of GFAP-positive astrocytes was significantly increased (9.25 ± 3.62, 10.13 ± 3.31, 29.25 ± 11.80, 19.75 ± 5.31 and 10.63 ± 3.02 cells/section in the Sham, 1 day, 3 day, 7 day and 28 day groups, respectively), astrocytic vessel coverage was not increased following CCH (astrocytic coverage(%): 33.21 ± 6.47, 30.87 ± 12.31, 32.79 ± 8.14, 42.60 ± 5.89 and 37.81 ± 6.05 in the Sham, 1 day, 3 day, 7 day and 28 day groups, respectively) (Additional file 1: Figure S5). This indicates that the role of astrocyte activation is increasing neuroinflammation, not promoting astrocytic coverage of the microvasculature following CCH.

### Transport of neurotoxic molecules through the BBB occurs via endothelial transcytosis following CCH

**Fig. 5:**
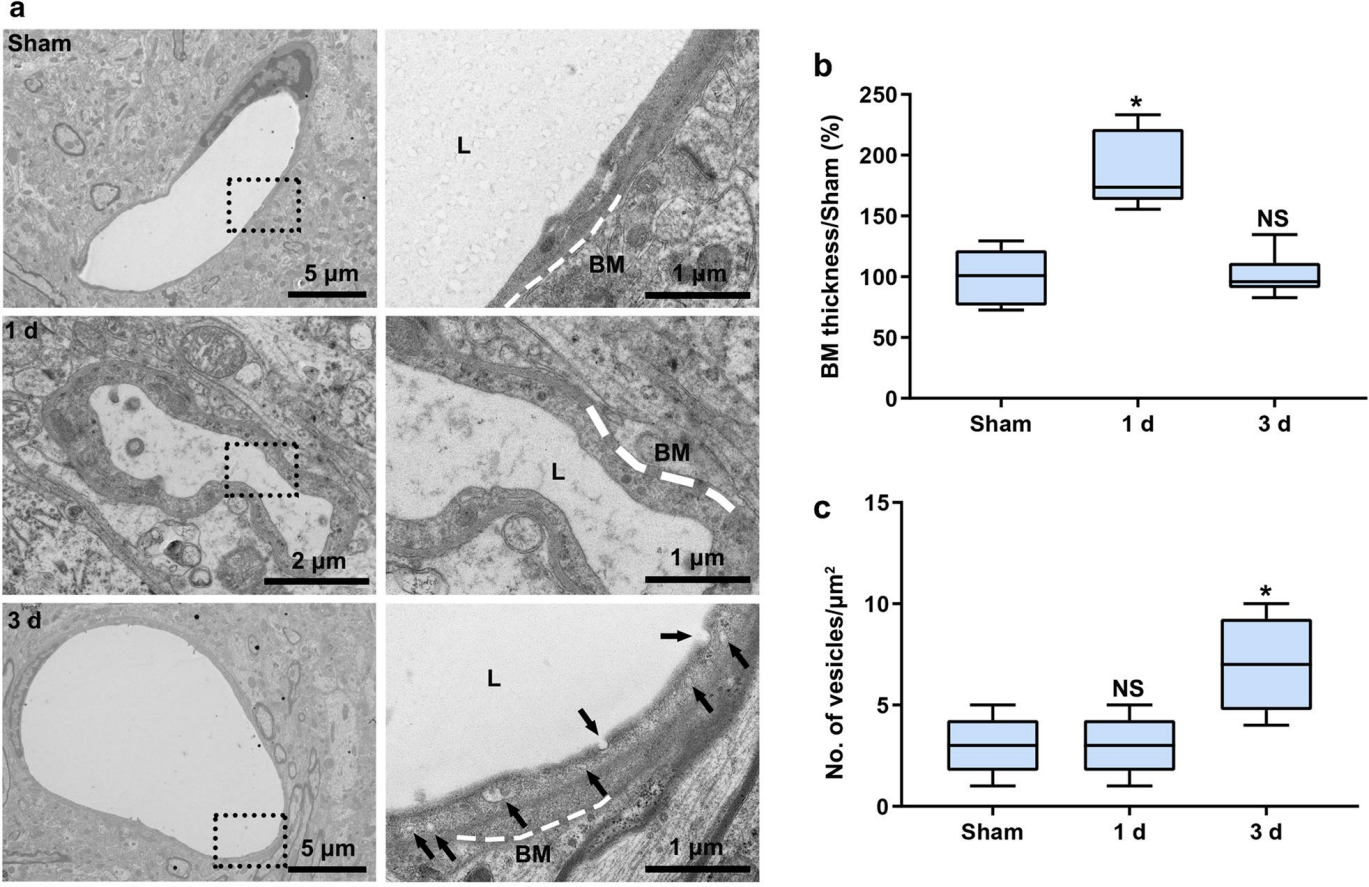
Increased endothelial transcytosis following CCH. **a** Representative images of the ultrastructure of the microvasculature 1 and 3 days postoperation. The white dotted lines indicate the BM of the vessel. The black arrows indicate vesicles in EC. L: lumen. **b, c** Quantification of the BM thickness and the number of vesicles in EC. n = 5 per group; NS, not significant; *p < 0.05 compared to the Sham group; one-way ANOVA followed by Dunnett’s post hoc test

We further used TEM to observe the ultrastructural changes of the BBB. We found that the microvasculature was edematous and that BM thickness was increased 1 day postoperation (BM thickness/Sham(%): 100.20 ± 22.39, 186.50 ± 31.60 and 101.00 ± 17.87 in the Sham, 1 day and 3 day groups, respectively) (Fig. 5a, b). Edema was decreased and BM thickness returned to the normal level, but vesicle density in EC was significantly increased 3 days postoperation (number of vesicles: 3.0 ± 1.41, 3.0 ± 1.41 and 7.0 ± 2.37/μm^2^ in the Sham, 1 day and 3 day groups, respectively) (Fig. 5a, c). CCH did not alter the ultrastructure of endothelial TJ (Additional file 1: Figure S6). These results indicate that large neurotoxic molecules enter the brain parenchyma through increased endothelial transcytosis.

### Molecular signatures of transcytosis increased following CCH

**Fig. 6:**
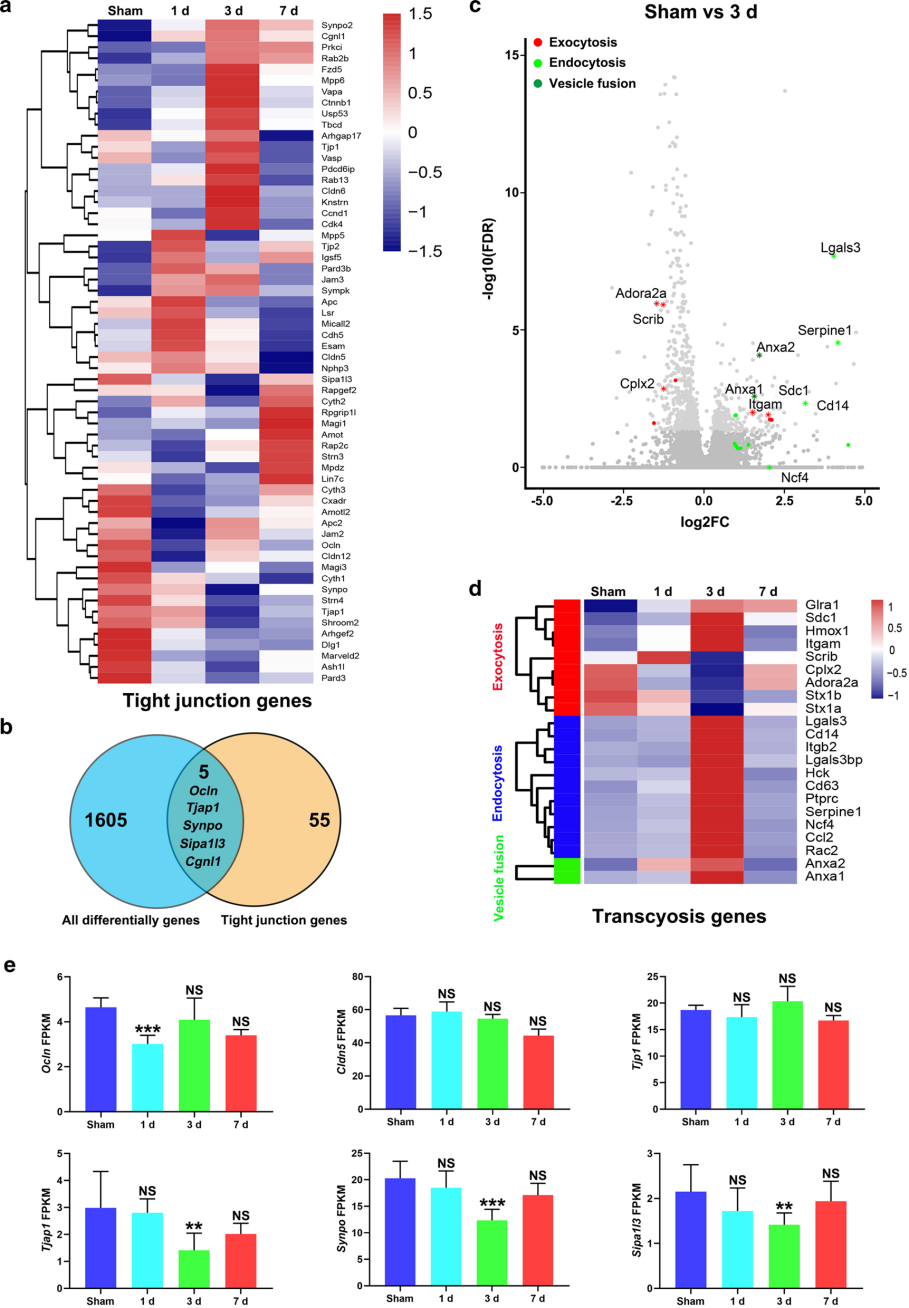
Increased transcytosis molecular signatures following CCH. **a** Heatmap of genes related to BBB TJ components in the Sham, 1 day, 3 day and 7 day groups. **b** Venn diagram of the number of all differentially expressed genes and BBB TJ components genes. All differentially genes represent different timepoints compared to the Sham group. **c **Volcano plot showing altered genes between the 3 day group and the Sham group. Light gray circles indicate differentially expressed genes. Significantly altered gene ontology (GO) terms are highlighted (red, exocytosis; light green, endocytosis; dark green, vesicle fusion). *selected genes of interest. **d** Heatmap of genes related to exocytosis, endocytosis and vesicle fusion in the Sham, 1 day, 3 day and 7 day groups. **e** Fragments per kilobase of transcript per million mapped reads (FPKM) of BBB TJ components related markers. n = 3 biologically independent samples per group; NS, not significant; **p < 0.01 and ***p < 0.001 compared to the Sham group; p values shown were derived from the DESeq2 statistical package

BBB integrity was impairment from 1 to 7 days following CCH. To further observe the transcriptional molecular changes of BBB associated biological properties, we performed bulk RNA sequencing of the CC at 1 day, 3 days and 7 days following CCH. Differentially expressed genes were most significantly changed 3 days postoperation (Additional file 1: Figure S7). Analysis of the differentially expressed transcripts revealed most of TJ components associated genes were not significantly changed at different timepoints (Fig. 6a, b), as shown the mRNA levels of occludin (*Ocln*), claudin 5 (*Cldn5*) and TJ protein 1 (*Tjp1*), three of the classical TJ components (Fig. 6e). Interestingly, we found signatures of exocytosis (e.g., *Cplx2*, *Adora2a*, *Itgam*, *Scrib*) was significantly altered 3 days postoperation (Fig. 6c). However, there were significant increase in signatures for endocytosis (e.g., *Serpine1*, *Lgals3* and *Cd14*) and vesicle fusion (e.g., *Anxa1*, *Anxa2*) were significantly increased 3 days postoperation (Fig. 6c, d). These results reflect that transcytosis associated molecular signatures are significantly increase, whereas TJ components associated molecular signature was not changed. BBB dysfunction may be mostly resulting from increased endothelial transcytosis rather than BBB TJ components.

### BBB dysfunction precedes neuroinflammation and demyelination following CCH

Neuroinflammation is an important factor in the pathogenesis of CSVD [33, 34]. The number of microglia reached its peak 3 days postoperation, and 86% of microglia were activated. From day 7 to 28 postoperation, the number of microglia decreased (7.60 ± 2.86, 11.84 ± 3.24, 41.12 ± 10.69, 14.20 ± 6.31 and 13.52 ± 6.38 cells/section in the Sham, 1 day, 3 day, 7 day and 28 day groups, respectively). However, 58% of microglial were still activated at 28 days postoperation (percentage of activated microglial: 23.32 ± 6.63, 32.62 ± 12.35, 87.08 ± 9.05, 61.23 ± 17.56 and 59.52 ± 16.07 in the Sham, 1 day, 3 day, 7 day and 28 day groups, respectively), indicating that neuroinflammation persisted until at least 28 days postoperation (Additional file 1: Figure S8).

WMLs is another core pathological change that occurs in CSVD [35]. The myelin sheath is formed by mature myelin-producing oligodendrocytes, and WM damage is caused by the loss of mature myelin-producing oligodendrocytes [36]. Using immunohistochemistry staining for MBP, a marker of the myelin sheath around neuronal axons, we found that the MBP density in the CC progressively decreased from 3 to 28 days postoperation (MBP density/Sham: 0.96 ± 0.10, 0.82 ± 0.14, 0.80 ± 0.12, 0.55 ± 0.12 and 0.58 ± 0.15 in the Sham, 1 day, 3 day, 7 day and 28 day groups, respectively) (Additional file 1: Figure S9A, B). MBP density was significantly different between the surgery group and the Sham group until 28 days postoperation (Additional file 1: Figure S9B). Western blotting analysis also showed that MBP protein levels were lower in the 28 day group than in the Sham group (MBP/β-actin: 1.64 ± 0.41, 1.50 ± 0.31, 1.37 ± 0.30, 0.54 ± 0.20 and 0.65 ± 0.22 in the Sham, 1 day, 3 day, 7 day and 28 day groups, respectively) (Additional file 1: Figure S9C, D). Although there was a slight increase in MBP protein levels at 56 days postoperation, there was no significant difference in levels between 56 days postoperation and 28 days postoperation. These results indicate that BBB breakdown precedes neuroinflammation and the formation of WMLs, and that BBB breakdown may therefore be a key pathological event following CCH.

### BBB dysfunction activates TGF-β signaling following CCH

**Fig. 7:**
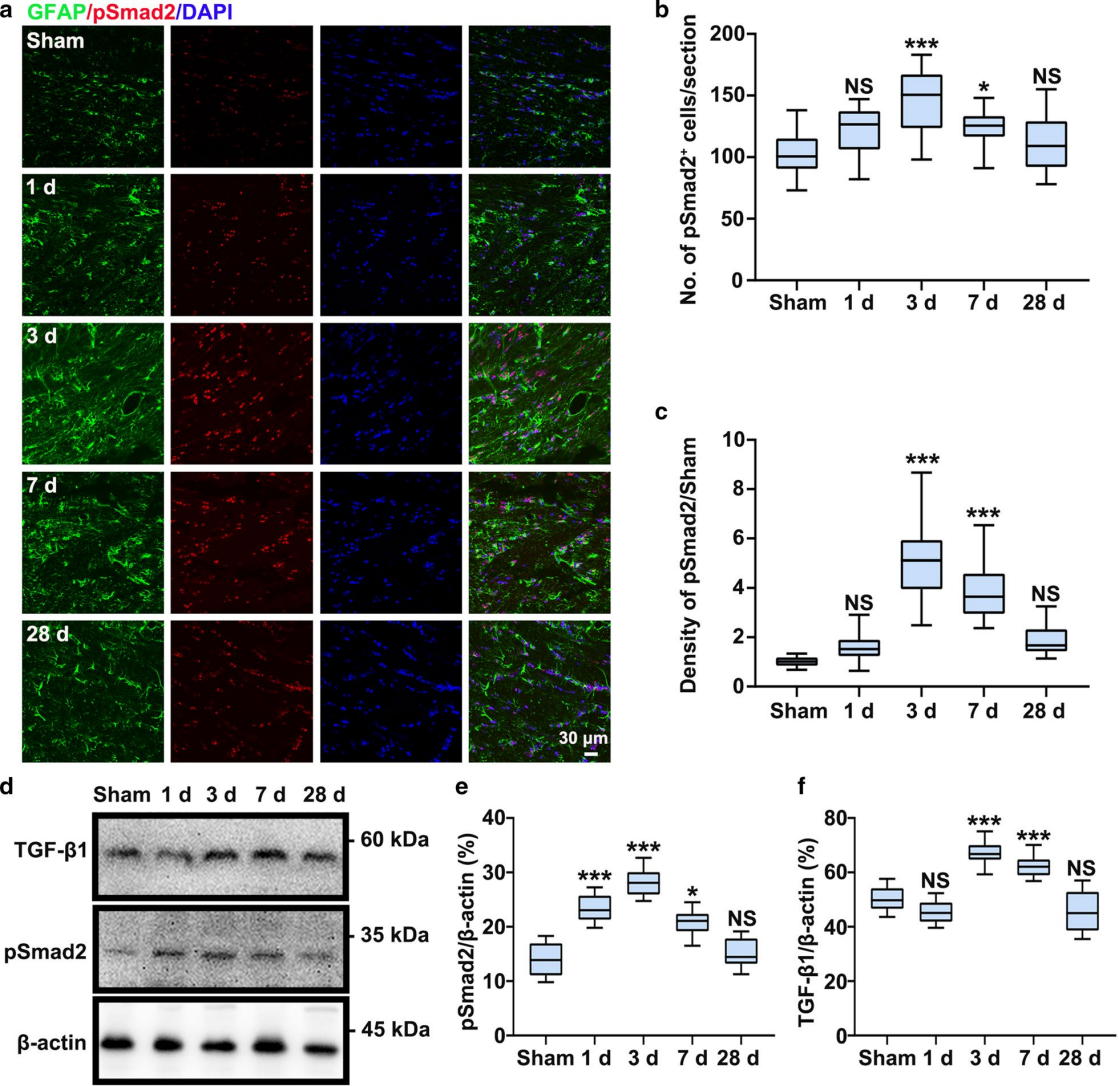
Activation of TGF-β signaling after BBB breakdown following CCH. **a** Immunofluorescence staining for GFAP and pSmad2 at different timepoints. **b, c** Quantification of pSmad2^+^ cells and the pSmad2 density in the CC at different timepoints. **d–f** Western blotting analysis and quantification of pSmad2 and TGF-β1 expression in the CC at different timepoints. n = 5 per group; NS, not significant; *p < 0.05 and ***p < 0.001 compared to the Sham group; one-way ANOVA followed by Dunnett’s post hoc test

After traumatic brain injury (TBI), serum protein leakage causes a robust injury response by activating the transforming growth factor-β (TGF-β) signaling pathway [37, 38]. Hence, we next investigated whether the TGF-β signaling pathway is also a candidate mechanism underlying brain injury following CCH (Fig. 7). Immunostaining for pSmad2, which is the downstream of the TGF-β receptor, revealed that the number of pSmad2-positive cells (102.30 ± 17.55, 120.60 ± 20.55, 145.40 ± 25.78, 123.70 ± 14.21 and 110.70 ± 21.65 cells/section in the Sham, 1 day, 3 day, 7 day and 28 day groups, respectively) and the pSmad2 density (pSmad2 density/Sham: 1.01 ± 0.20, 1.62 ± 0.56, 5.22 ± 1.64, 3.84 ± 1.10 and 1.88 ± 0.61 in the Sham, 1 day, 3 day, 7 day and 28 day groups, respectively) were significantly increased in the 3 day group compared with the Sham group (Fig. 7a–c). Using Western blotting, we further found increased concentrations of pSmad2 (pSmad2/β-actin(%): 14.03 ± 2.97, 23.50 ± 2.51, 28.26 ± 2.48, 20.85 ± 2.34 and 15.06 ± 2.59 in the Sham, 1 day, 3 day, 7 day and 28 day groups, respectively) and TGF-β1 (TGF-β1/β-actin(%): 50.13 ± 4.34, 45.40 ± 4.13, 67.15 ± 4.56, 62.10 ± 3.93 and 45.40 ± 7.46 in the Sham, 1 day, 3 day, 7 day and 28 day groups, respectively) following CCH (Fig. 7d–f). This result indicates that the change in TGF-β signaling activation is consistent with BBB breakdown. The consequences of BBB breakdown are regulated by TGF-β signaling following CCH.

### Maintenance of BBB integrity ameliorates brain damage following CCH

**Fig. 8:**
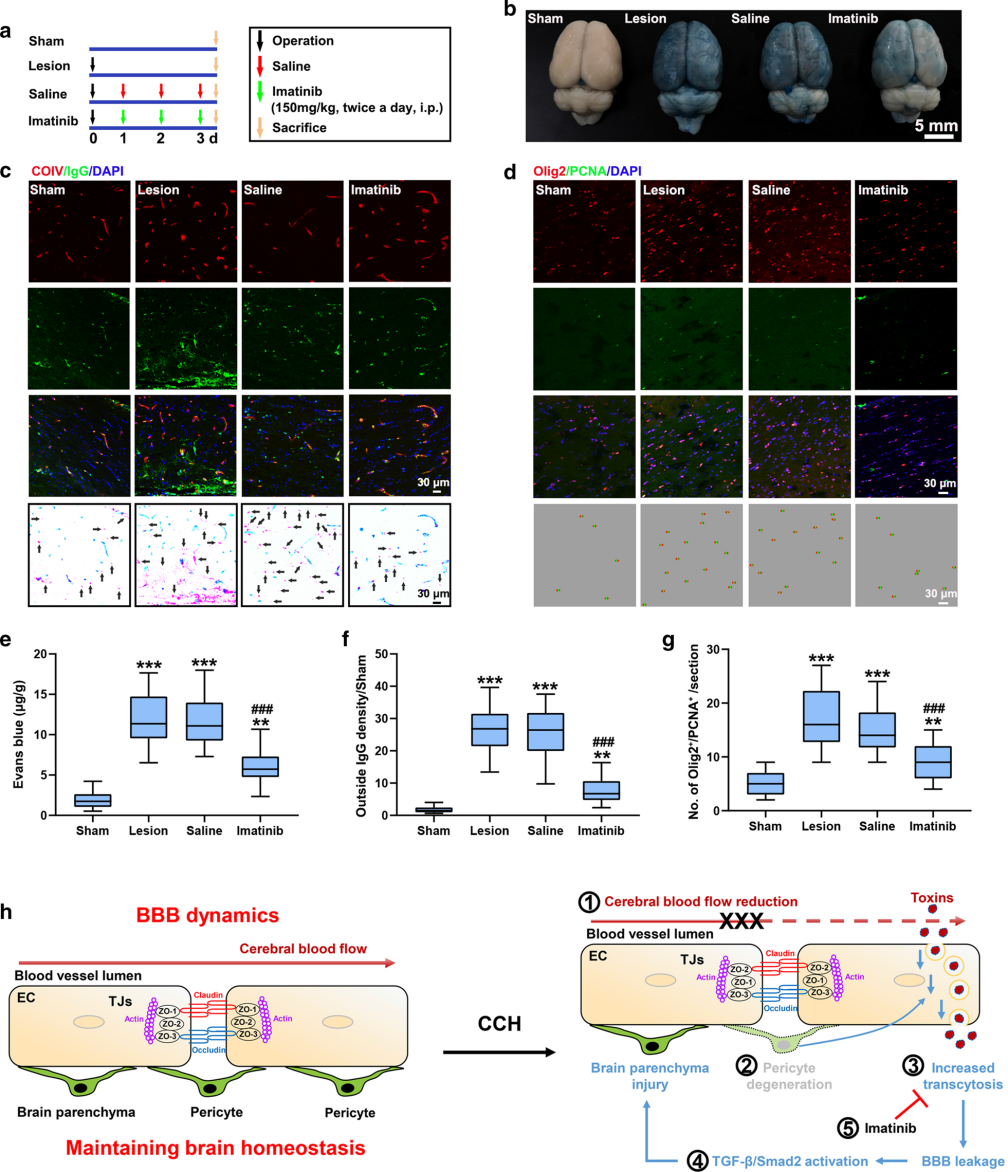
Extravasation of EB and IgG is abolished after imatinib treatment. **a** Schematic representation of the procedures of imatinib treatment. **b** Gross anatomic changes in the Sham, Lesion, Saline and Imatinib groups after EB injection. **c** Confocal images showing reduced IgG extravasation in the CC after imatinib treatment. A schematic of IgG in a representative confocal microscopy image is shown in the right column. The black arrows indicate IgG accumulation outside of the vessels. **d** Confocal images showing reduced OPC activation in the CC after imatinib treatment. **e–g** Quantification of EB leakage, outside IgG density, and the number of proliferative OPC (Olig2^+^PCNA^+^) in the CC after imatinib treatment. **h** Schematic representation of the cascade of pathological changes following CCH. n = 10 per group; **p < 0.01 and ***p < 0.001 compared to the Sham group; ^###^p < 0.001 compared to the Saline group; one-way ANOVA followed by Dunnett’s post hoc test

Neurotoxic molecules enter the brain parenchyma via increased endothelial transcytosis following CCH. Imatinib can effectively decrease BBB permeability by blocking the signaling of platelet-derived growth factor receptor alpha (PDGFR-α) in ischemic stroke [19]. Thus, we assessed the role of imatinib treatment on BBB integrity and neural function following CCH. We found that imatinib treatment significantly reduced EB leakage (EB concentration: 1.87 ± 0.93, 11.99 ± 3.28, 11.72 ± 3.24 and 5.97 ± 1.86 μg/g in the Sham, Lesion, Saline and Imatinib groups, respectively) and brain IgG accumulation (outside IgG density/Sham: 1.73 ± 0.97, 26.35 ± 6.98, 25.31 ± 7.48 and 7.72 ± 3.87 in the Sham, Lesion, Saline and Imatinib groups, respectively) (Fig. 8a–c, e, f). After imatinib treatment, vesicle density in EC was also significantly decreased (number of vesicles: 2.6 ± 1.78, 8.3 ± 1.95, 8.4 ± 2.01 and 5.1 ± 2.33/μm^2^ in the Sham, Lesion, Saline and Imatinib groups, respectively) (Additional file 1: Figure S10). This indicates that imatinib effectively maintains BBB integrity following CCH. We further assessed pathological outcomes after imatinib treatment. We found that the number of proliferative OPC (4.93 ± 2.15, 17.03 ± 5.39, 15.00 ± 4.35 and 9.13 ± 3.25 in the Sham, Lesion, Saline and Imatinib groups, respectively) were decreased after imatinib treatment (Fig. 8d, g). The percentage of activated microglia (29.17 ± 5.32, 66.35 ± 9.58, 65.35 ± 11.35 and 40.45 ± 7.68 in the Sham, Lesion, Saline and Imatinib groups, respectively) and TGF-β signaling were also decreased (pSmad2 density/Sham: 1.05 ± 0.33, 5.42 ± 1.09, 4.90 ± 0.96 and 3.96 ± 1.10 in the Sham, Lesion, Saline and Imatinib groups, respectively) (Additional file 1: Figure S11A, B, D, E). Whereas, the PDGFR-β protein levels were not significantly changed after imatinib treatment (PDGFR-β/β-actin (%): 53.48 ± 6.29, 31.85 ± 5.01, 35.08 ± 5.14 and 35.80 ± 4.86 in the Sham, Lesion, Saline and Imatinib groups, respectively) (Additional file 1: Figure S11C, F). The results indicate that BBB dysfunction is directly involved in the regulation of neuroinflammatory responses and OPC proliferation by regulating TGF-β signaling. Imatinib exerts its protective effect on BBB integrity through endothelial transcytosis rather than through the regulation of pericyte loss.

## Discussion

CSVD refers to a group of pathological changes with various aetiologies, including age, hypertension, heredity and others [4]. All these factors can affect CBF [5]. While it has been found that CCH and BBB dysfunction are the common pathophysiology in different types of CSVD [10, 39], which BBB component becomes impaired, how neurotoxic molecules are able to enter into the parenchyma, and whether maintenance of BBB integrity can be used as a treatment strategy for CSVD are unclear. WMLs, in contrast, are well understood to be the hallmark of CSVD pathology [10, 35]. The molecular mechanisms that lead to brain parenchymal damage after BBB breakdown following CCH have not yet been properly examined. In this study, we observed the timeline of both BBB dysfunction and the formation of WMLs following CCH. We revealed that BBB leakage occurs earlier than other pathological events, including OPC activation, mature oligodendrocyte loss, astrocytic activation, and microglial activation, following CCH. Furthermore, we thoroughly examined the components of the BBB. The key change in BBB components following CCH is pericyte loss, which is apparently the leading cause of BBB impairment. Blood-derived pathogens enter into the brain parenchymal through increased endothelial transcytosis rather than endothelial paracellular passage. TGF-β signaling regulates the consequences of BBB breakdown following CCH. Our findings suggest that disease development resulting from CCH unfolds as follows (Fig. 8h). CCH leads to a reduction in pericyte coverage, which induces increased BBB permeability. Following BBB impairment, blood-derived neurotoxic substances enter the brain parenchyma via endothelial endocytosis. Blood-derived neurotoxic substances initiate the inflammatory response, OPC activation and other pathological events by regulating the TGF-β/pSmad2 signaling pathway. Ultimately, homeostasis of oligodendroglial lineage cells is disturbed, resulting in the formation of irreversible WMLs. Furthermore, imatinib treatment ameliorated endothelial endocytosis and brain parenchyma injury. Together, these findings demonstrate that BBB impairment plays a predominant regulatory role in the occurrence of brain damage following CCH, suggesting that BBB compromise is the primary driving factor leading to progressive neural dysfunction. Reversal of BBB dysfunction may be a promising strategy for treating CSVD.

The BBB limits the free diffusion of molecules from the blood into the parenchymal to maintain brain microenvironment homeostasis [31, 40]. BBB dysfunction contributes to the pathology of many neurological diseases, including TBI [37], stroke [41], AD [17], aging [42] and CSVD [10]. Recent studies have shown that BBB dysfunction in the hippocampus occurs earlier than cognitive impairment in AD [43]. BBB dysfunction is an early biomarker of AD [43]. On the other hand, during normal aging, WM integrity is maintained after BBB impairment [44]. These results suggest that BBB dysfunction occurs in the early stage of neurological diseases and that BBB impairment is the key factor leading to brain parenchymal injury. In this study, we found that BBB impairment precedes a series of pathological events, including astrocyte activation, microglial activation, OPC activation and the formation of WMLs. BBB dysfunction is the link between blood-derived pathogens and neural dysfunction.

The cellular components of the BBB include EC and pericyte [31]. It has been reported that EC dysfunction is the primary cause of BBB dysfunction in stroke-prone spontaneously hypertensive rats [45]. This rat model is known to be a model of human sporadic CSVD [46]. On the other hand, Ding et al. analyzed the frontal WM in the postmortem brains from 124 subjects with poststroke dementia (PSD), VD, AD, or AD-VD (mixed), poststroke nondemented (PSND) stroke survivors and normal aging controls [47]. The researchers found that capillary pericyte loss was a common characteristic among these patients [47]. Ding et al. indicated that capillary pericyte loss is the structural basis of BBB dysfunction in aging-related dementias [47]. Furthermore, Bell et al. also found that pericytes control key neurovascular functions in the adult brain and during normal aging [48]. Our study revealed that CCH does not alter the microvascular number or endothelial TJ expression of claudin-5 and ZO-1. While microvascular length, occludin protein expression, and pericyte coverage are reduced following CCH, microvascular diameter and BM thickness are increased. Pericyte loss may be the leading cause of BBB impairment, and other structural changes may be secondary to pericyte loss following CCH. First, there is a strong positive correlation between pericyte loss and BBB permeability. Second, significant loss of pericytes occurs from 1 day postoperation, i.e., in the early stage of CCH. Most of the other structural changes occur from 3 days postoperation. Third, it has been reported that pericytes play a critical role in maintaining BBB integrity [27, 29]. Therefore, on the basis of previous studies and our study, we can infer that the structural bases of BBB impairment in a variety of neurodegenerative diseases are somewhat similar. This finding has important implications for the development of new therapeutic strategies.

EC is key plays in direct communication between the blood and the brain parenchyma. EC exhibits two distinctive features in maintaining BBB integrity [28]. One is a specialized TJ that blocks paracellular passage between the blood and the brain parenchyma [11]. The other is exhibited unusually low levels of transcytotic vesicles that limit transcellular transport [49, 50]. Our results show that TJ is not damaged and endothelial endocytosis is increased following CCH. In addition, BBB TJ molecular signatures are not significantly altered. Whereas, transcytosis molecular signatures are significantly increased. This indicates that blood-derived pathogens enter the brain parenchyma through increased endothelial endocytosis after pericyte loss. This finding is consistent with the results of previous research. Using many model of adult viable pericyte loss, Armulik et al. also found that pericytes maintain BBB integrity via transcytosis [27]. On the other hand, pericyte loss occurs as early as 1 days postoperation. OPC, astrocytic, microglial and TGF-β signaling activation are followed by pericyte loss. Until 28 days postoperation, the homeostasis of oligodendroglial lineage cells are disrupted resulting in irreversible WMLs. It was also demonstrated that CCH induced neuronal cell death was gradually progressed with time in the CA1 subfield of hippocampus [18]. At 12 weeks following CCH, it exhibited obviously neuronal injury in the CA1 [20]. Although pericyte loss is a transient process and can be recovered spontaneously following CCH, brain parenchymal injury caused by pericyte loss is persistent.

Although little is known about the regulatory pathways that trigger brain parenchymal injury after BBB breakdown in CSVD, the relevant molecular mechanisms that regulate brain damage in other neurological diseases involving BBB dysfunction can provide some clues. Most studies on the interaction between blood-derived proteins and neural structure have involved AD [17], TBI [38] and aging [42]. The TGF-β signaling pathway regulates progressive neural dysfunction after BBB breakdown in TBI and aging [38, 42]. However, the brain region of interest in TBI and aging is not the CC. TBI and aging also have different pathologies [51]. However, although the triggers of mouse models of stroke, multiple sclerosis, TBI and seizure are different, they all involve profound BBB disruption [52]. Interestingly, EC RNA sequencing revealed similar gene expression changes in EC in these four diseases [52]. We also examined whether the TGF-β signaling pathway is the mechanism that leads to brain damage following CCH. We found that TGF-β/pSmad2 signaling is promoted after BBB breakdown following CCH. An increase in TGF-β/pSmad2 signaling is responsible for brain damage. Reversal of BBB dysfunction ameliorates TGF-β/pSmad2 signaling activation and brain damage. Therefore, the TGF-β/pSmad2 signaling pathway regulates brain parenchymal injury after BBB breakdown following CCH. These studies also suggest that although BBB dysfunction is triggered by distinct factors in different neurological disorders, the regulatory pathways leading to neurovascular dysfunction after BBB breakdown exhibit similar responses.

BBB disruption is associated with severe brain parenchyma injury following CCH. Increased BBB permeability involves various components [11]. Thus, currently, there is no well documented therapeutic drugs for BBB integrity maintenance. However, imatinib has shown promising therapeutic candidate drug to restore BBB integrity. Imatinib inhibits several tyrosine kinases and is primarily used to treat chronic myeloid leukemia and gastrointestinal stromal tumor [53]. Apart from its well documented antitumor applications, imatinib also has been demonstrated its therapeutic effect in a range of neurological diseases including autoimmune encephalomyelitis, ischemic stroke, brain hemorrhage, multiple sclerosis, Parkinson’s disease, AD, Huntington’s disease and spinal cord injury [53]. The therapeutic target of imatinib is different in these conditions. Furthermore, study has demonstrated imatinib binds to phosphorylation sites on the PDGFR-α and blocks PDGFR-α signaling to maintain BBB integrity in model of ischemic stroke [19]. In the clinical trial, Wahlgren et al. did not observe any serious adverse events in acute ischemic stroke patients treated with imatinib and imatinib treatment improved neurological and functional outcomes [54]. Armulik et al. also found imatinib blocks endothelial endocytosis in models of pericyte loss [27]. In our study, we find imatinib treatment reduces BBB permeability. In addition, imatinib executes a protective role on neural function following CCH. It also should be note that imatinib treatment for BBB protection is very different from antitumor long-term applications. There will be more studies to assess the dose and time requirement for its unusual usage following CCH.

It should be noted that although our findings suggest that a reduction in pericyte coverage leads to BBB dysfunction through increased endothelial transcytosis following CCH, it is not clear why EC, which represent a direct link between blood and neural function, are not the primary cause of BBB dysfunction [55]. However, BBB dysfunction is related to EC transcytosis after pericyte loss following CCH. Therefore, pericyte and EC are closely linked in BBB function. In future studies, we plan to isolate brain microvessel fragments from the CC and then generate single-cell suspensions for single-cell RNA sequencing. Furthermore, we will use single-cell analysis to study the cause of pericyte loss, the response of EC after pericyte loss and the crosstalk between pericyte and EC. Ultimately, we will analyze the relationship between pericyte and EC in health and disease.

## Conclusions

In summary, our findings show that pericytes regulate BBB permeability and the formation of WMLs following CCH. We found that loss of pericyte coverage leads to BBB dysfunction and accumulation of neurotoxic molecules. Neurotoxic molecules enter the brain parenchyma via endothelial endocytosis. BBB dysfunction triggers neuroinflammation and other pathological events, leading to increased OPC proliferation, reduced OPC maturation and, eventually, the death of mature oligodendrocytes and the development of WMLs. BBB protection ameliorates neurotoxic molecule accumulation and decreases OPC activation. Thus, our findings have important implications for understanding the pathogenesis of CSVD and suggest that loss of pericyte coverage is a key trigger. With these insights, potential therapeutic strategies for CSVD can be developed.

## Supplementary Information

**Additional file 1: Figure S1. Accumulation of EB in the whole brain following CCH.** (A) Representative gross anatomic images of brain tissues (up) and corresponding coronal sections (down) cutting along the white dotted line in the Sham group and the 3 day group after EB injection. (B) Quantification of EB leakage in the cortex, CC and striatum. n = 10 per group; ***p < 0.001 compared to the Sham group; two-way ANOVA. **Figure S2. Accumulation of plasma protein in the CC following CCH.** (A) Triple staining for COIV (red), IgG (green) and DAPI (blue) for assessment of IgG leakage in the CC. A schematic of IgG in representative confocal microscopy image is shown in the right column. The black arrows indicate extravascular IgG deposits. (B and C) Quantification of total IgG and IgG leakage in the CC at different timepoints. n = 8 per group; NS, not significant; *p < 0.05, **p < 0.01 and ***p < 0.001 compared to the Sham group; one-way ANOVA followed by Dunnett’s post hoc test. **Figure S3. Microvascular changes in the CC following CCH.** (A) Immunofluorescence staining for Glut1 (green) for assessment of capillary changes following CCH. (B and C) Quantification of the number of capillary and capillary diameter in the CC following CCH. n = 8 per group; NS, not significant; **p < 0.01 compared to the Sham group; one-way ANOVA followed by Dunnett’s post hoc test. **Figure S4. BBB TJ changes in the CC following CCH.** (A-C) Western blotting analysis occludin, claudin 5 and ZO-1 expression in the CC at different timepoints. (D-F) Quantification of occludin, claudin 5 and ZO-1 expression in the CC at different timepoints. n = 5 per group; NS, not significant; *p < 0.05 compared to the Sham group; one-way ANOVA followed by Dunnett’s post hoc test. **Figure S5. Immunofluorescence assessment of vessels covered by astrocytes in the CC following CCH.** (A) Confocal images of immunofluorescence staining for GFAP (green) and COIV (red) in CC. (B and C) Quantification of the number of astrocytes cells (GFAP^+^) and the percentage of vessels covered by astrocytes in the CC at different timepoints. n = 8 per group; NS, not significant; *p < 0.05 and ***p < 0.001 compared to the Sham group; one-way ANOVA followed by Dunnett’s post hoc test. **Figure S6. Endothelial junctions changes following CCH.** Representative images of the ultrastructure of endothelial junctions 1 and 3 days postoperation. The black arrows indicate endothelial junctions. **Figure S7. Transcriptional changes following CCH.** (A) Bar graphs showing the number of upregulated and downregulated expressed genes at each timepoints following CCH. (B) Venn diagram of the number of differentially expressed genes between different timepoints. **Figure S8. Activation of microglia in the CC following CCH.** (A) Immunofluorescence staining for Iba1 (green) for evaluation of microglial activation following CCH. (B and C) Quantification of the total number of microglia (Iba1^+^) and the percentage of activated microglia in the CC at different timepoints. n = 8 per group; NS, not significant; *p < 0.05 and ***p < 0.001 compared to the Sham group; one-way ANOVA followed by Dunnett’s post hoc test. **Figure S9. Progressively demyelination in the CC after CCH.** (A and B) Immunofluorescence staining and quantification of MBP density in the CC at different timepoints after operation. (C and D) Western blotting analysis and quantification of MBP expression in the CC at different timepoints. n = 5 per group; NS, not significant; ***p < 0.001 compared to the Sham group; one-way ANOVA followed by Dunnett’s post hoc test. **Figure S10. Endothelial transcytosis is arrested after imatinib treatment.** (A) Representative images of the ultrastructure of the microvasculature in the Sham, Lesion, Saline and Imatinib groups. The black arrows indicate vesicles in EC. L: lumen. (B) Quantification of the number of vesicles in EC. n = 5 per group; *p < 0.05 and **p < 0.01 compared to the Sham group; #p < 0.05 compared to the Saline group; one-way ANOVA followed by Dunnett’s post hoc test. **Figure S11. Imatinib treatment ameliorates microglial activation and aberrant TGF-β/Smad2 signaling activation.** (A and B) Confocal images showing reduced microglial activation (A) and pSmad2 density (B) in the CC after imatinib treatment. (C) Western blotting analysis of PDGFR-β expression in the CC after imatinib treatment. (D-F) Quantification of percentage of activated microglial cells, pSmad2 density and PDGFR-β expression in the CC after imatinib treatment. n = 10 per group; **p < 0.01 and ***p < 0.001 compared to the Sham group; ###p < 0.001 compared to the Saline group; one-way ANOVA followed by Dunnett’s post hoc test.

## Data Availability

All data generated and analyzed during the study are included in this published article.

## Abbreviations

2VO: Permanent bilateral common carotid artery occlusion
AD: Alzheimer’s disease
ALB: Albumin
BBB: Blood–brain barrier
BM: Basement membrane
CC: Corpus callosum
CCH: Chronic cerebral hypoperfusion
CNS: Central nervous system
CSVD: Cerebral small vessel disease
EB: Evans blue
EC: Endothelial cell
MBP: Myelin basic protein
OPC: Oligodendrocyte progenitor cell
PDGFR: Platelet-derived growth factor receptor
PDGFR-α: Platelet-derived growth factor receptor alpha
PDGFR-β: Platelet-derived growth factor receptor beta
PSD: Post-stroke dementia
PSND: Post-stroke non-demented
TBI: Traumatic brain injury
TEM: Transmission electron microscopy
TGF-β: Transforming growth factor-β
TJ: Tight junction
VD: Vascular dementia
WMHs: White matter hyperintensities
WMLs: White matter lesions
