# Supplementary material for: Reduction in pericyte coverage leads to blood–brain barrier dysfunction via endothelial transcytosis following chronic cerebral hypoperfusion
Source: Fluids Barriers CNS. 2021 May 5;18:21. doi: 10.1186/s12987-021-00255-2 (PMC8101037; doi:10.1186/s12987-021-00255-2)
Supplement: Supplementary file 1 — Additional file 1: Figure S1. Accumulation of EB in the whole brain following CCH. (A) Representative gross anatomic images of brain tissues (up) and corresponding coronal sections (down) cutting along the white dotted line in the Sham group and the 3 day group after EB injection. (B) Quantification of EB leakage in the cortex, CC and striatum. n = 10 per group; ***p < 0.001 compared to the Sham group; two-way ANOVA. Figure S2. Accumulation of plasma protein in the CC following CCH. (A) Triple staining for COIV (red), IgG (green) and DAPI (blue) for assessment of IgG leakage in the CC. A schematic of IgG in representative confocal microscopy image is shown in the right column. The black arrows indicate extravascular IgG deposits. (B and C) Quantification of total IgG and IgG leakage in the CC at different timepoints. n = 8 per group; NS, not significant; *p < 0.05, **p < 0.01 and ***p < 0.001 compared to the Sham group; one-way ANOVA followed by Dunnett’s post hoc test. Figure S3. Microvascular changes in the CC following CCH. (A) Immunofluorescence staining for Glut1 (green) for assessment of capillary changes following CCH. (B and C) Quantification of the number of capillary and capillary diameter in the CC following CCH. n = 8 per group; NS, not significant; **p < 0.01 compared to the Sham group; one-way ANOVA followed by Dunnett’s post hoc test. Figure S4. BBB TJ changes in the CC following CCH. (A-C) Western blotting analysis occludin, claudin 5 and ZO-1 expression in the CC at different timepoints. (D-F) Quantification of occludin, claudin 5 and ZO-1 expression in the CC at different timepoints. n = 5 per group; NS, not significant; *p < 0.05 compared to the Sham group; one-way ANOVA followed by Dunnett’s post hoc test. Figure S5. Immunofluorescence assessment of vessels covered by astrocytes in the CC following CCH. (A) Confocal images of immunofluorescence staining for GFAP (green) and COIV (red) in CC. (B and C) Quantification of the number of astrocytes ce [file 12987_2021_255_MOESM1_ESM.docx]

Additional file for

**Reduction in pericyte coverage leads to blood-brain barrier dysfunction via endothelial transcytosis following chronic cerebral hypoperfusion**

Zhengyu Sun^1†^, Chenhao Gao^1†^, Dandan Gao^1^, Ruihua Sun^1^, Wei Li^1^, Fengyu Wang^1^, Yanliang Wang^2^, Huixia Cao^2^, Guoyu Zhou^3^, Jiewen Zhang^1*^, Junkui Shang^1*^

^1^Department of Neurology, Henan Provincial People’s Hospital, Zhengzhou University People’s Hospital, Henan University People’s Hospital, Zhengzhou, Henan, 450003, China.

^2^Department of Nephrology, Henan Provincial Key Laboratory of Kidney Disease and Immunology, Henan Provincial People's Hospital, Zhengzhou University People’s Hospital, Henan University People’s Hospital, Zhengzhou, Henan, 450003, China.

^3^School of Public Health, Zhengzhou University, Zhengzhou, Henan, 450001, China.

*Corresponding author. E-mail: zhangjiewen9900@126.com (Jiewen Zhang), shangjunkui@yeah.net (Junkui Shang)

†These authors contributed equally to this work.

**Additional file 1: Figures**


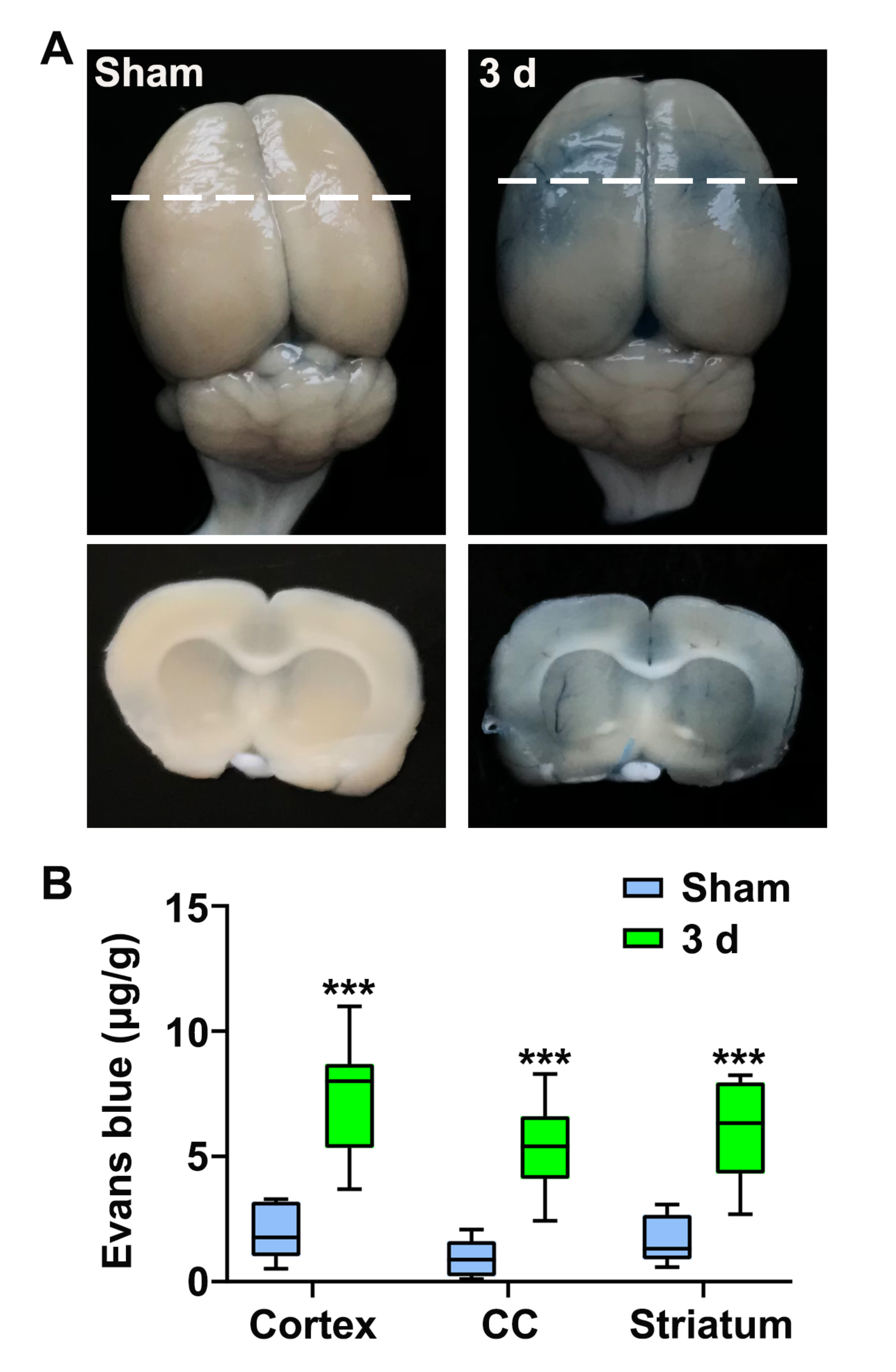


**Additional file 1: Figure S1. Accumulation of EB in the whole brain following CCH.** (A) Representative gross anatomic images of brain tissues (up) and corresponding coronal sections (down) cutting along the white dotted line in the Sham group and the 3 day group after EB injection. (B) Quantification of EB leakage in the cortex, CC and striatum. n=10 per group; ***p < 0.001 compared to the Sham group; two-way ANOVA.


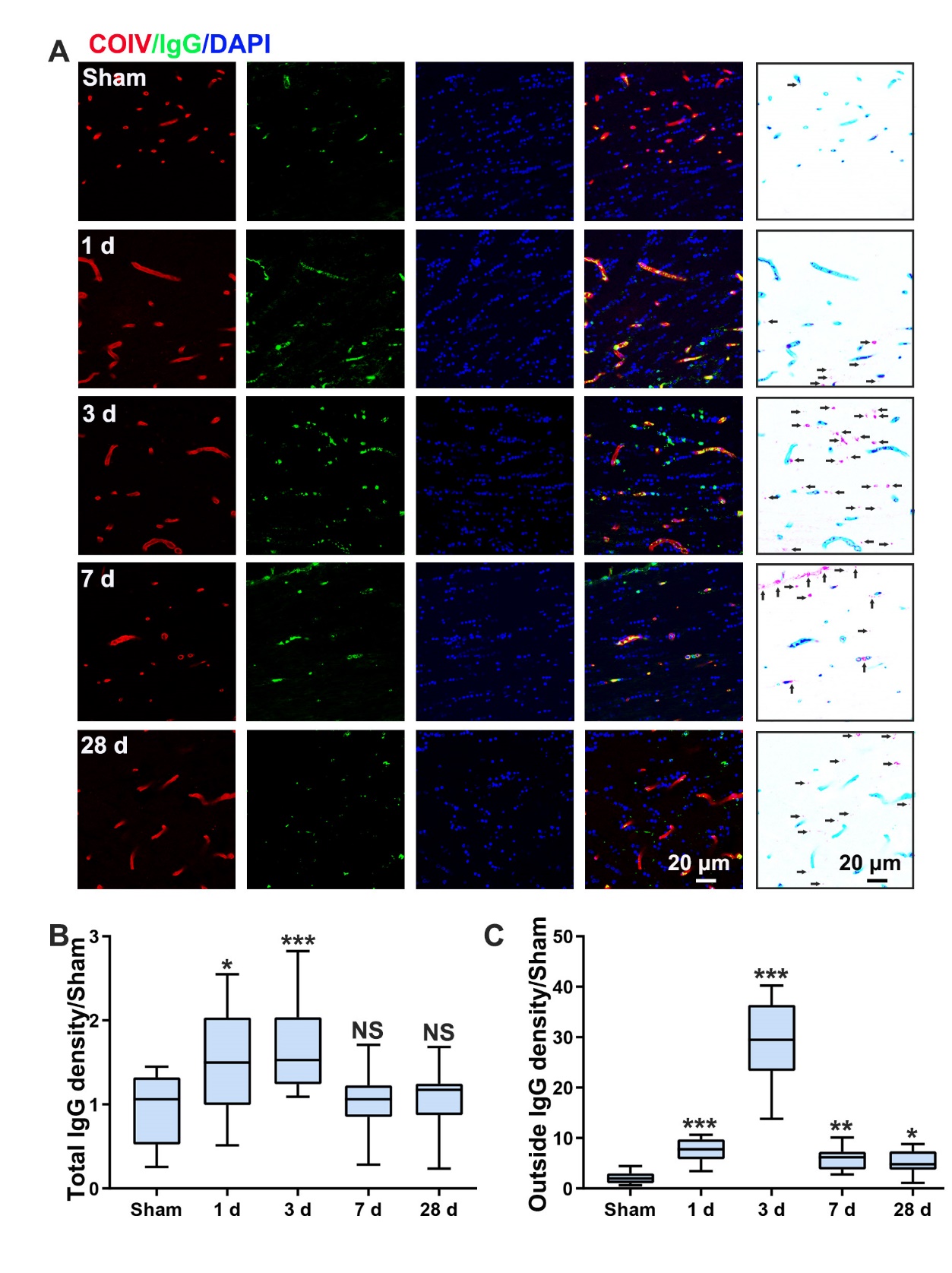


**Additional file 1: Figure S2. Accumulation of plasma protein in the CC following CCH.** (A) Triple staining for COIV (red), IgG (green) and DAPI (blue) for assessment of IgG leakage in the CC. A schematic of IgG in representative confocal microscopy image is shown in the right column. The black arrows indicate extravascular IgG deposits. (B and C) Quantification of total IgG and IgG leakage in the CC at different timepoints. n=8 per group; NS, not significant; *p < 0.05, **p < 0.01 and ***p < 0.001 compared to the Sham group; one-way ANOVA followed by Dunnett’s post hoc test.


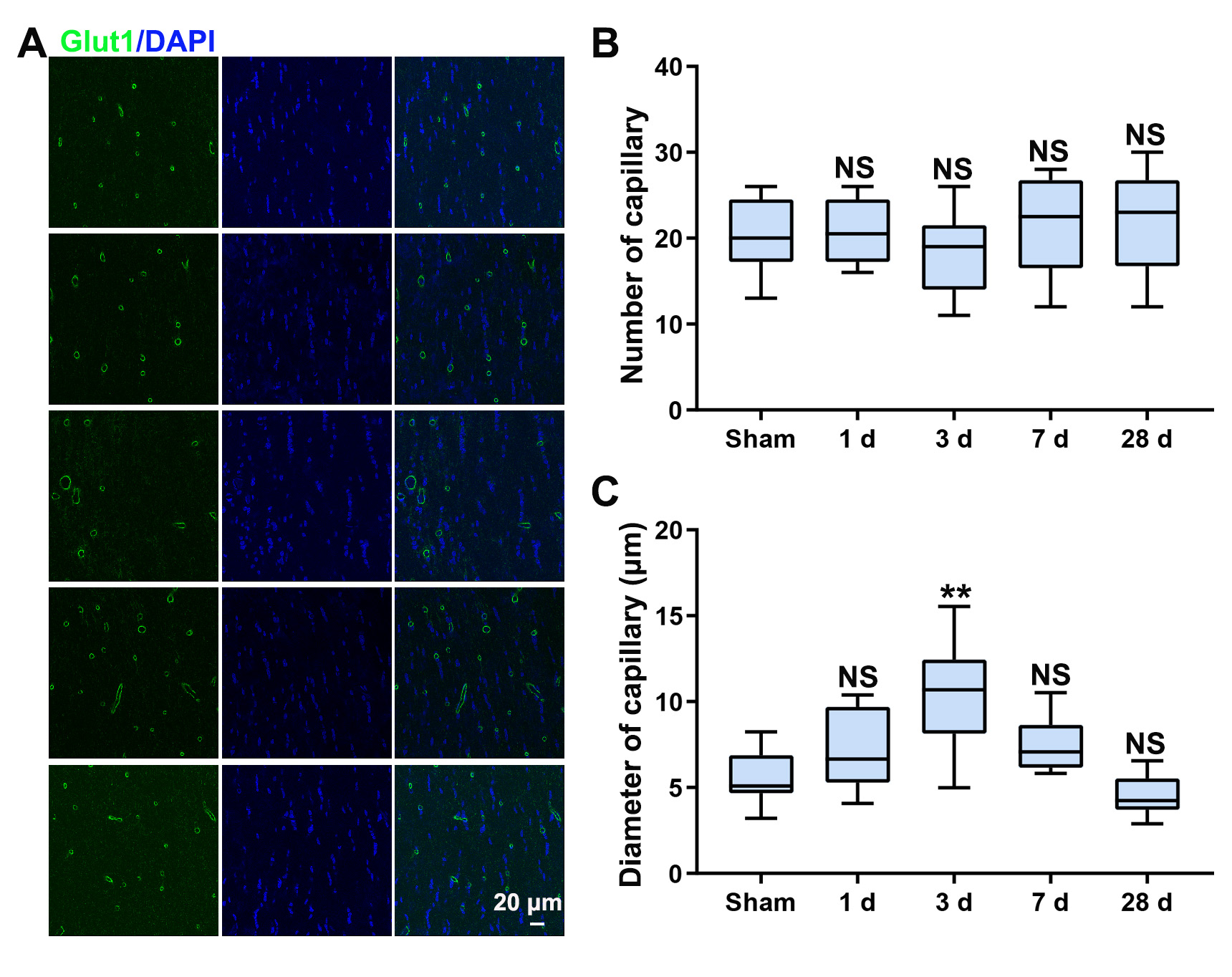


**Additional file 1: Figure S3. Microvascular changes in the CC following CCH.** (A) Immunofluorescence staining for Glut1 (green) for assessment of capillary changes following CCH. (B and C) Quantification of the number of capillary and capillary diameter in the CC following CCH. n=8 per group; NS, not significant; **p < 0.01 compared to the Sham group; one-way ANOVA followed by Dunnett’s post hoc test.


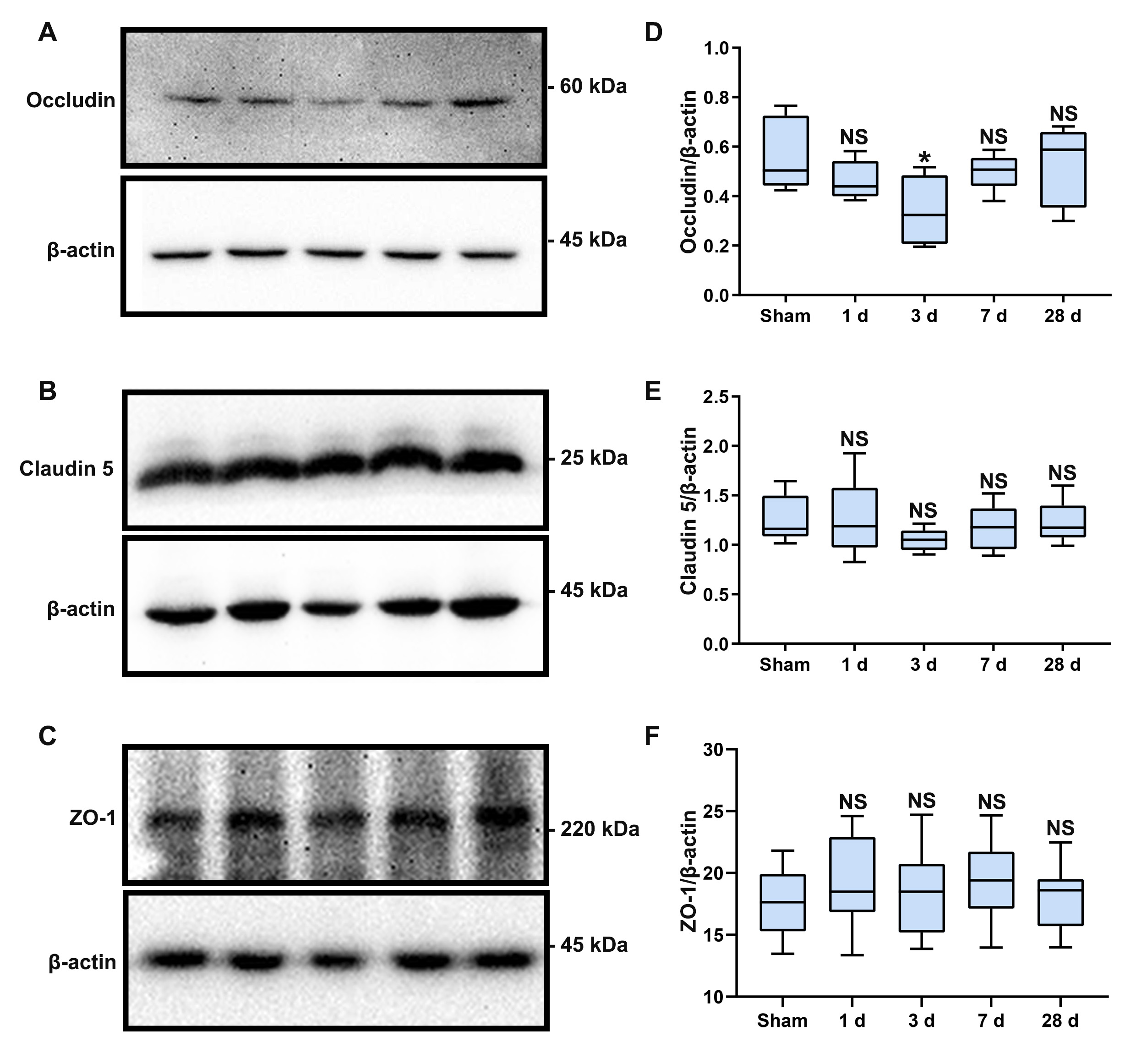


**Additional file 1: Figure S4. BBB TJ changes in the CC following CCH.** (A-C) Western blotting analysis occludin, claudin 5 and ZO-1 expression in the CC at different timepoints. (D-F) Quantification of occludin, claudin 5 and ZO-1 expression in the CC at different timepoints. n=5 per group; NS, not significant; *p < 0.05 compared to the Sham group; one-way ANOVA followed by Dunnett’s post hoc test.


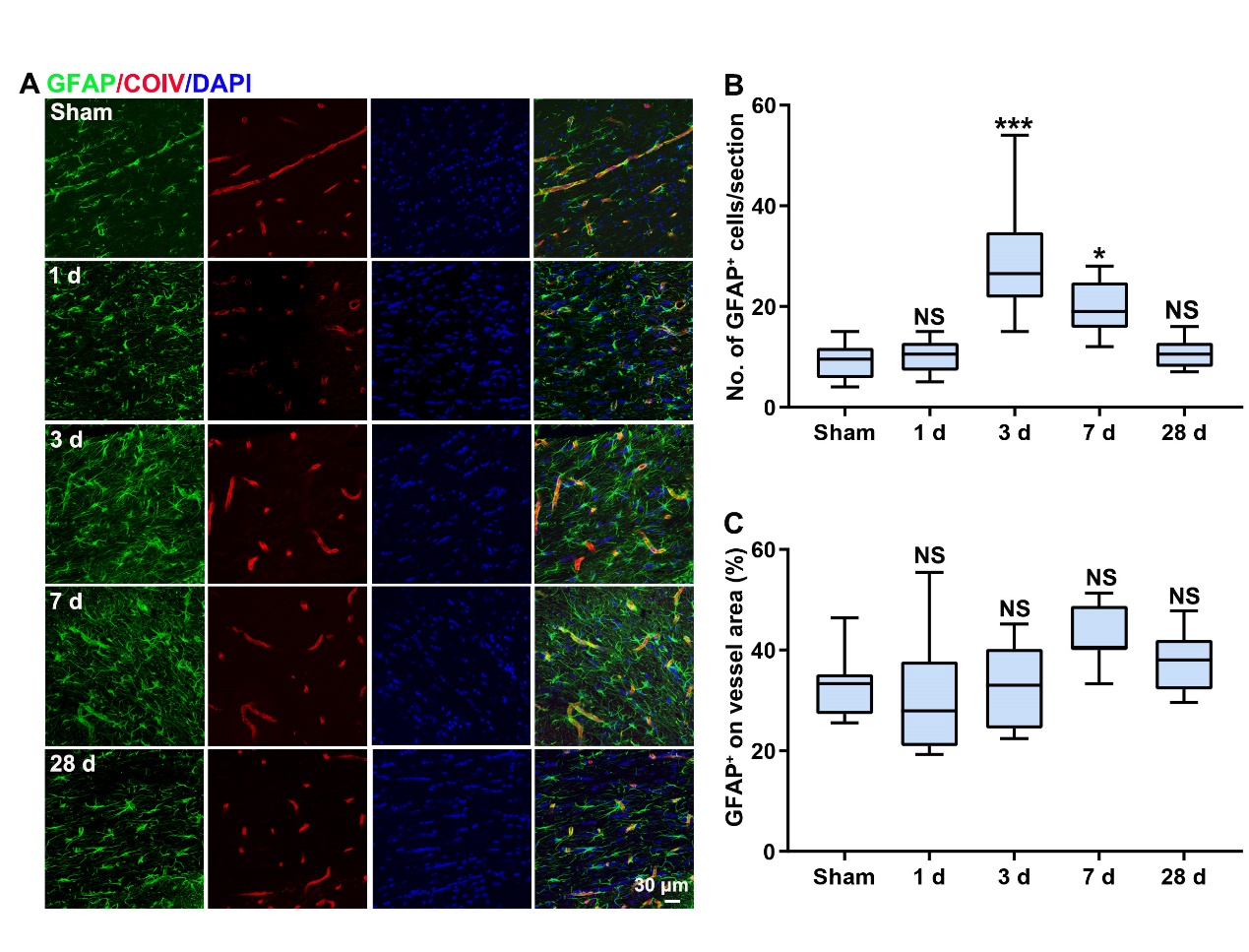


**Additional file 1: Figure S5. Immunofluorescence assessment of vessels covered by astrocytes in the CC following CCH.** (A) Confocal images of immunofluorescence staining for GFAP (green) and COIV (red) in CC. (B and C) Quantification of the number of astrocytes cells (GFAP^+^) and the percentage of vessels covered by astrocytes in the CC at different timepoints. n=8 per group; NS, not significant; *p < 0.05 and ***p < 0.001 compared to the Sham group; one-way ANOVA followed by Dunnett’s post hoc test.


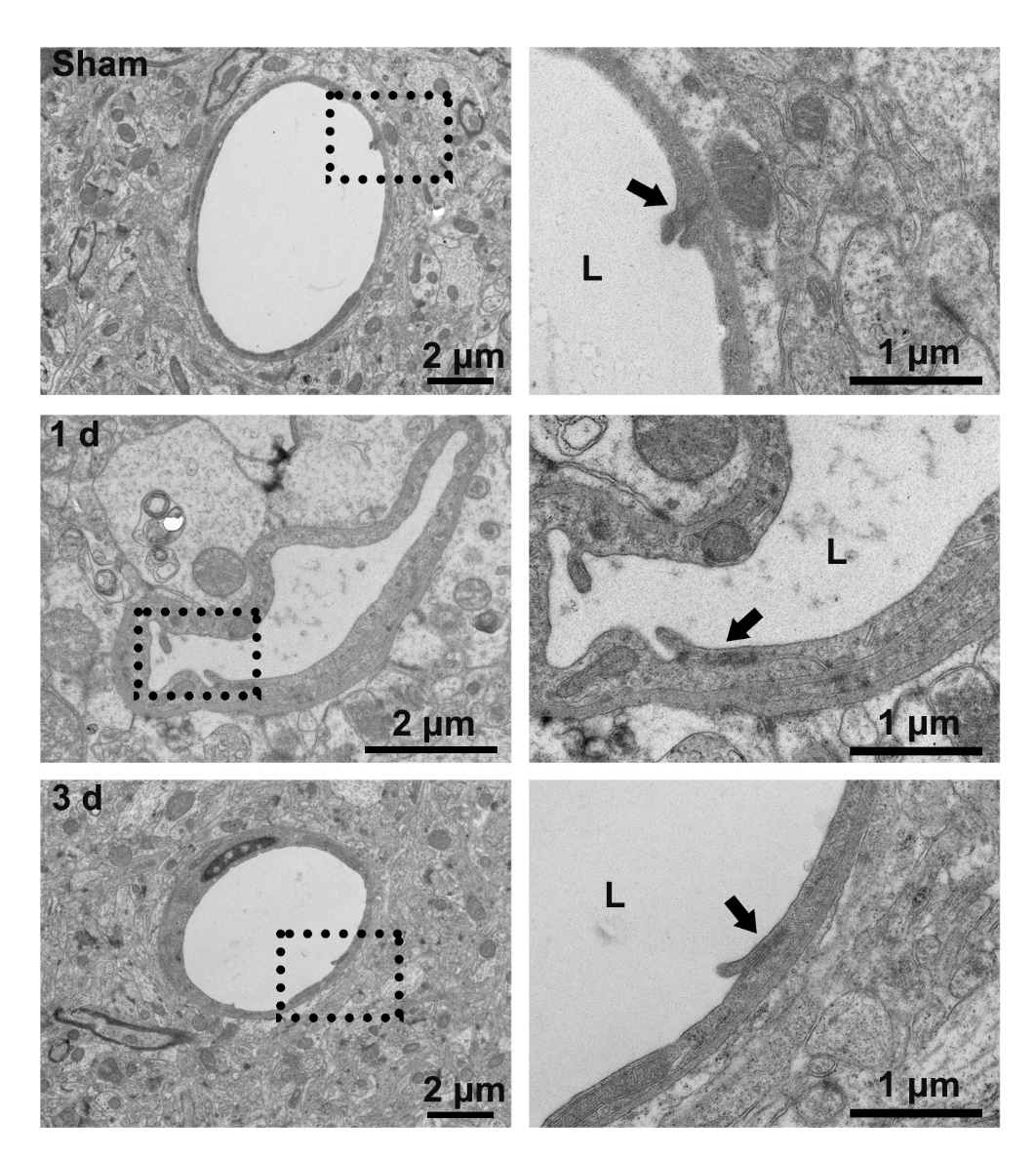


**Additional file 1: Figure S6. Endothelial junctions changes following CCH.** Representative images of the ultrastructure of endothelial junctions 1 and 3 days postoperation. The black arrows indicate endothelial junctions.


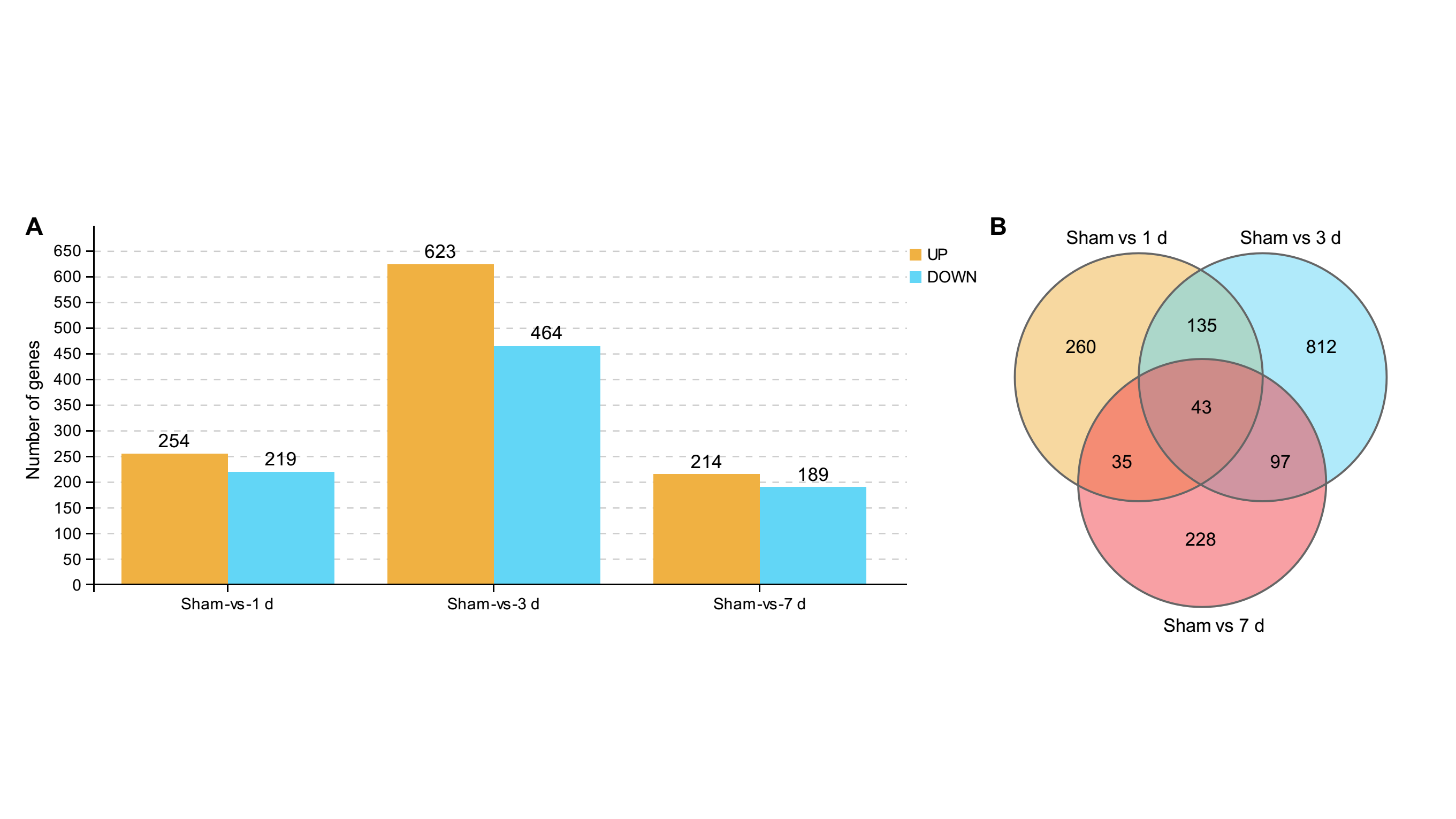


**Additional file 1: Figure S7. Transcriptional changes following CCH.** (A) Bar graphs showing the number of upregulated and downregulated expressed genes at each timepoints following CCH. (B) Venn diagram of the number of differentially expressed genes between different timepoints.


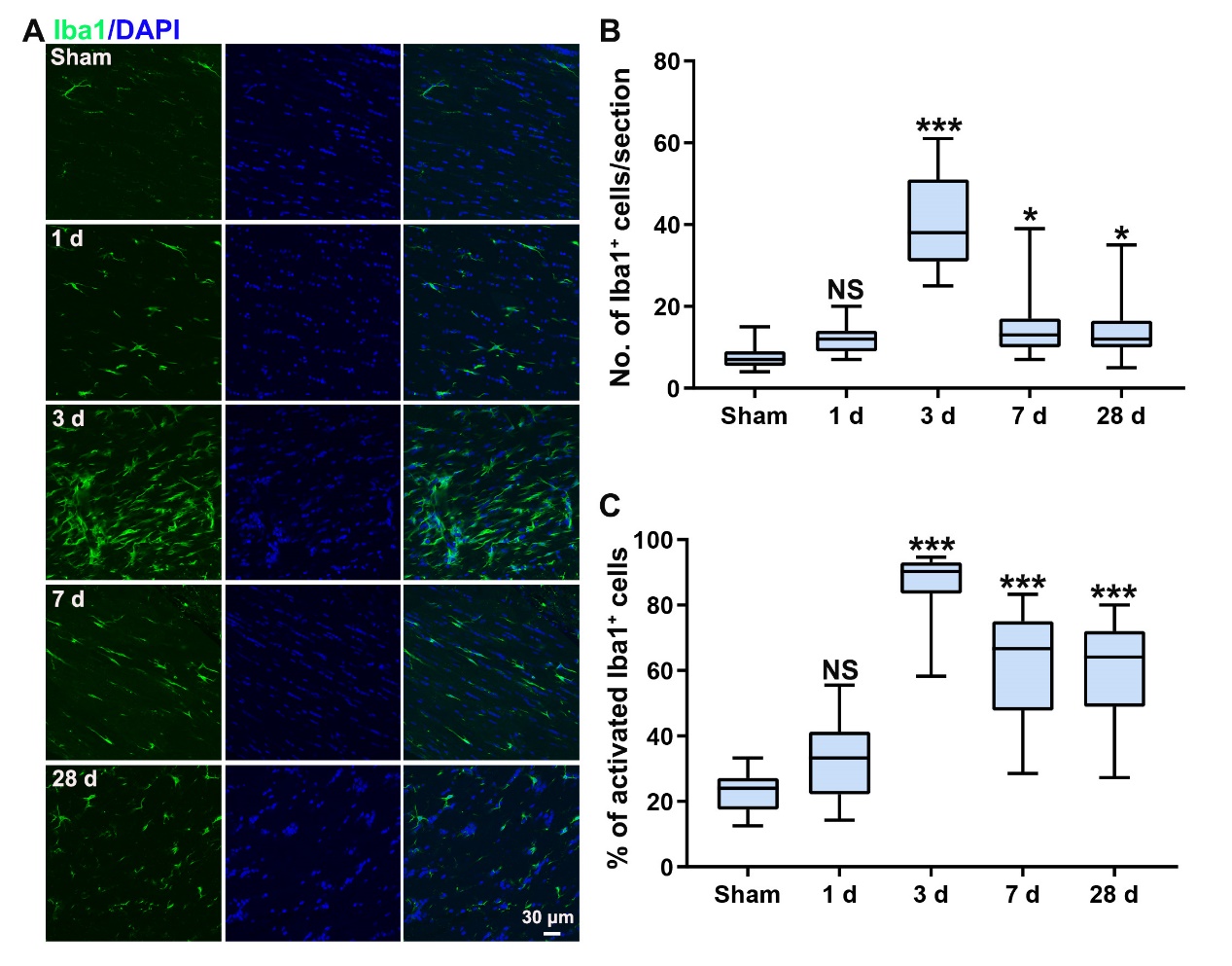


**Additional file 1: Figure S8. Activation of microglia in the CC following CCH.** (A) Immunofluorescence staining for Iba1 (green) for evaluation of microglial activation following CCH. (B and C) Quantification of the total number of microglia (Iba1^+^) and the percentage of activated microglia in the CC at different timepoints. n=8 per group; NS, not significant; *p < 0.05 and ***p < 0.001 compared to the Sham group; one-way ANOVA followed by Dunnett’s post hoc test.


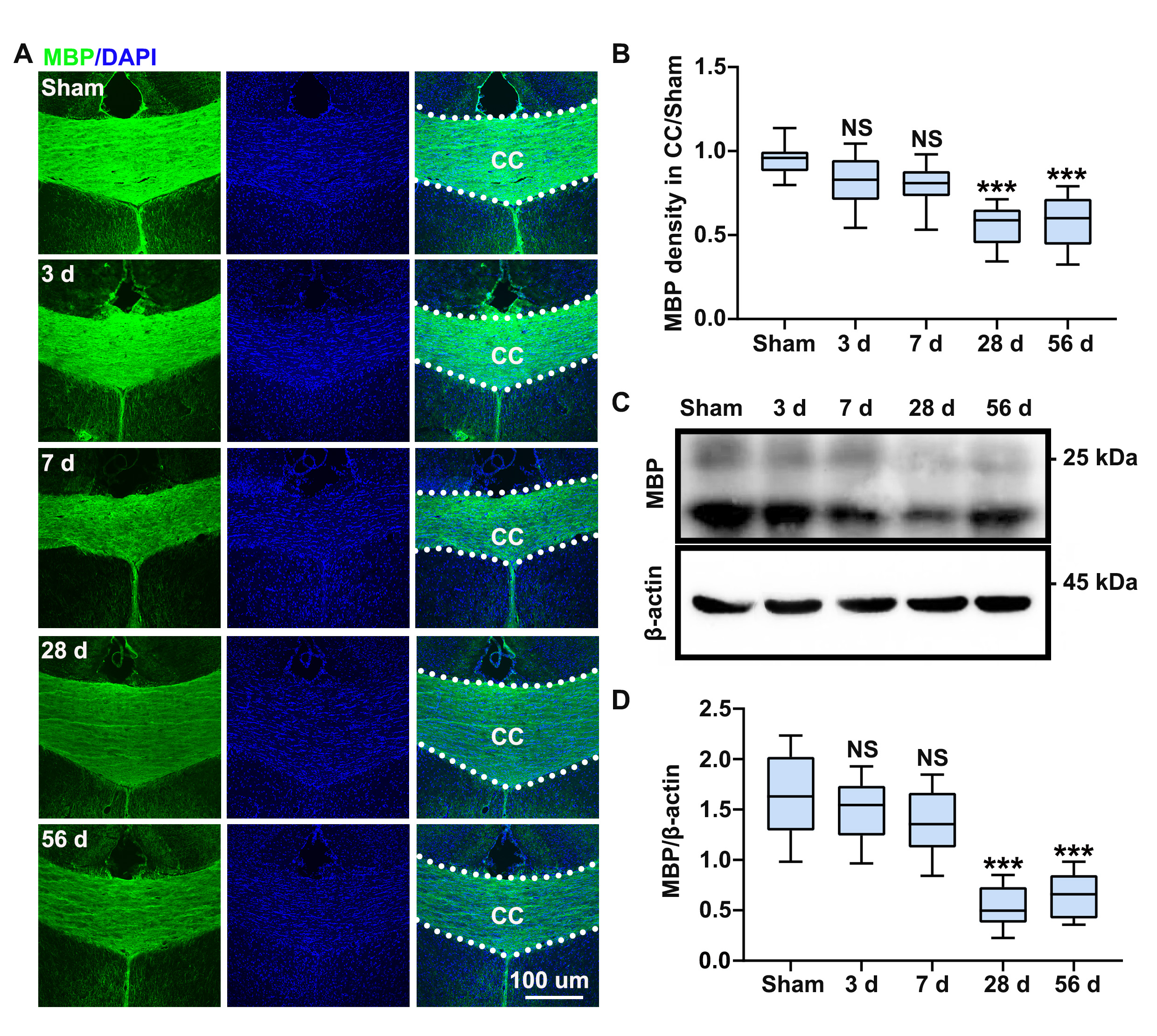


**Additional file 1: Figure S9. Progressively demyelination in the CC after CCH.** (A and B) Immunofluorescence staining and quantification of MBP density in the CC at different timepoints after operation. (C and D) Western blotting analysis and quantification of MBP expression in the CC at different timepoints. n=5 per group; NS, not significant; ***p < 0.001 compared to the Sham group; one-way ANOVA followed by Dunnett’s post hoc test.


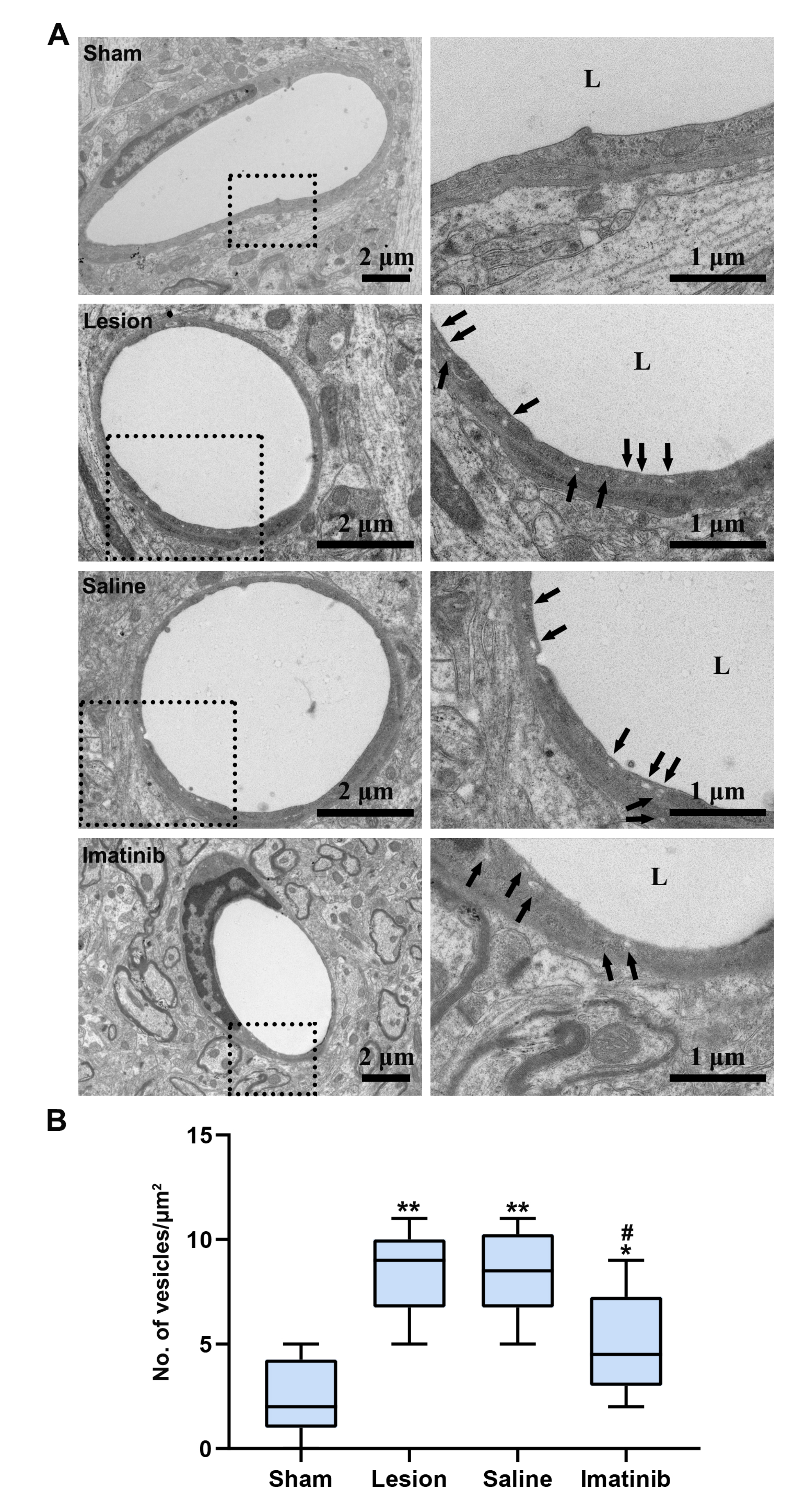


**Additional file 1: Figure S10. Endothelial transcytosis is arrested after imatinib treatment.** (A) Representative images of the ultrastructure of the microvasculature in the Sham, Lesion, Saline and Imatinib groups. The black arrows indicate vesicles in EC. L: lumen. (B) Quantification of the number of vesicles in EC. n=5 per group; *p < 0.05 and **p < 0.01 compared to the Sham group; #p < 0.05 compared to the Saline group; one-way ANOVA followed by Dunnett’s post hoc test.


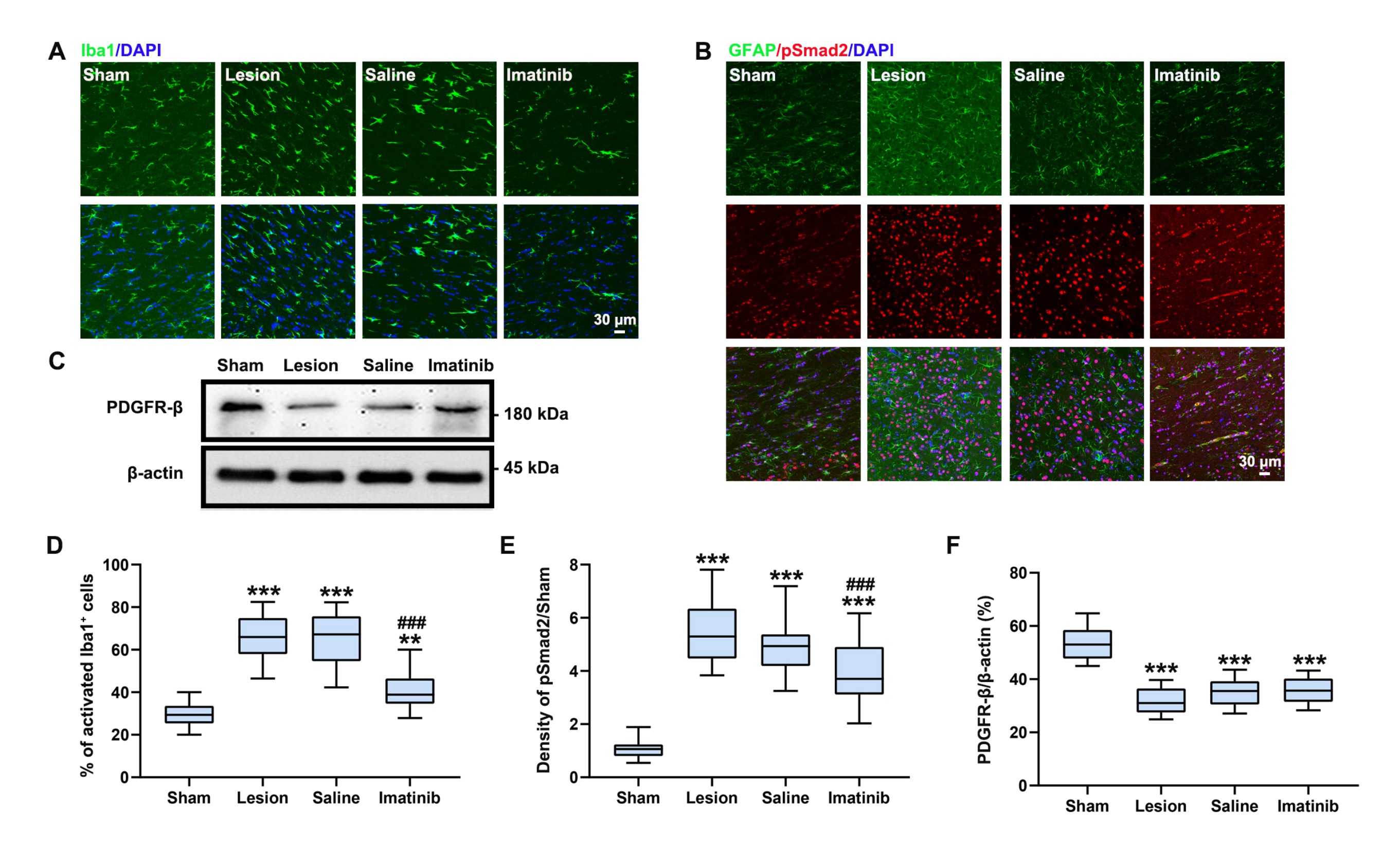


**Additional file 1: Figure S11. Imatinib treatment ameliorates microglial activation and aberrant TGF-β/Smad2 signaling activation.** (A and B) Confocal images showing reduced microglial activation (A) and pSmad2 density (B) in the CC after imatinib treatment. (C) Western blotting analysis of PDGFR-β expression in the CC after imatinib treatment. (D-F) Quantification of percentage of activated microglial cells, pSmad2 density and PDGFR-β expression in the CC after imatinib treatment. n=10 per group; **p < 0.01 and ***p < 0.001 compared to the Sham group; ###p < 0.001 compared to the Saline group; one-way ANOVA followed by Dunnett’s post hoc test.
